# Salidroside and Hongjingtian Injection Inhibit the Onset and Progression of Asthma via Pyroptosis in the Ozone‐Exposed Inflammation Environment

**DOI:** 10.1155/mi/9618148

**Published:** 2026-05-07

**Authors:** Zhidan Lu, Jingwen Li, Shujie Hou, Huiran Zhang, Xixin Yan

**Affiliations:** ^1^ The First Department of Pulmonary and Critical Care Medicine, The Second Hospital of Hebei Medical University, No. 215 Heping West Road, Shijiazhuang, Hebei, China, hebmu.edu.cn; ^2^ Hebei Key Laboratory of Respiratory Critical Care Medicine, Shijiazhuang, Hebei, China; ^3^ Hebei Institute of Respiratory Diseases, Shijiazhuang, Hebei, China; ^4^ The First Affiliated Hospital of Dalian Medical University, Dalian, Liaoning, China, dlmedu.edu.cn; ^5^ The Key Laboratory of Neural and Vascular Biology, Ministry of Education, Shijiazhuang, Hebei, China, moe.edu.cn; ^6^ Department of Medical and Pharmaceutical Informatics, Hebei Medical University, Shijiazhuang, Hebei, China, hebmu.edu.cn

**Keywords:** asthma, drug targets, inflammation, pyroptosis, therapeutic mechanisms

## Abstract

**Background:**

Ozone is a common air pollutant, and exposure to high concentrations of ozone can promote the onset and progression of asthma. Pyroptosis is a form of cell death associated with asthma exacerbation.

**Methods:**

This study aimed to identify ozone‐related pyroptosis genes through network toxicology and RNA‐seq analysis and to investigate the role of pyroptosis genes in asthma using multiple asthma tissue samples and machine learning methods. Key pyroptosis genes with significant roles in asthma were identified through experiments and multiomics approaches. Using network pharmacology methods, we screened traditional Chinese medicine (TCM) components associated with key pyroptosis genes. Through molecular clustering and molecular docking, we explored the interaction between TCM components and key genes. Using animal model drug interventions, we further analyzed the therapeutic effects of TCM components targeting pyroptosis on asthma under ozone exposure.

**Results:**

This study identified 16 ozone‐related pyroptosis genes through network pharmacology and transcriptomics data analysis. Based on multiple asthma tissue samples and machine learning methods, the 16 pyroptosis genes were found to have diagnostic predictive roles in asthma. Through experimentation and multiomics approaches, gasdermin D (GSDMD) was identified as a key gene in exacerbating asthma under ozone exposure. Furthermore, scRNA‐seq and virtual knockout revealed that GSDMD exerts a pro‐inflammatory effect on the asthma microenvironment. Using network pharmacology methods, we screened for salidroside, a Chinese herbal component related to GSDMD. Through molecular clustering and molecular docking, we identified seven salidroside analogs in Hongjingtian injection (HJT). Using an animal model drug intervention, we further analyzed the intervention effects of salidroside and HJT on asthma under ozone exposure.

**Conclusions:**

Ozone promotes the onset and progression of asthma by exacerbating inflammation and pyroptosis. salidroside and its analogs can target GSDMD to inhibit asthma exacerbation in ozone‐induced inflammation environments.

## 1. Introduction

Asthma is a common respiratory disease. Approximately 360 million people worldwide suffer from asthma, and the number of patients continues to grow [1]. Its characteristics include chronic airway inflammation, airway hyperresponsiveness, reversible airflow limitation, and structural changes in the airways that develop over time. The onset of asthma is closely associated with environmental factors. Allergens, infections, climate changes, physical activity, air pollution, smoking, and certain medications may also trigger asthma.

Ozone is an allotropic form of oxygen, appearing as a pale blue gas with a distinctive odor at room temperature. As one of the primary air pollutants, ozone pollution has been worsening globally [2]. In China, the scope and severity of ozone pollution have been increasing, with a rapid rise and spread [3–5]. Numerous epidemiological studies have shown that ozone increases the risk of asthma attacks, with this risk increasing as ozone concentrations rise, leading to significantly worsened asthma symptoms [6, 7], a significant increase in emergency department visits among asthma patients [8], and reduced responsiveness to rescue medications and increased risk of hospitalization among asthma patients [9, 10]. A Chinese epidemiological study [11] collected ozone exposure data from 4467 asthma patients in 18 Chinese cities, finding that short‐term exposure to ozone increases the risk of asthma onset, particularly among males and the elderly. Another study [12] involving 7270 asthma patients demonstrated that prolonged exposure to high levels of environmental ozone exacerbates asthma and may synergistically promote asthma progression in conjunction with sulfur dioxide.

Pyroptosis is a form of programmed cell death primarily mediated by the formation and activation of the NOD‐like receptor protein 3 (NLRP3) inflammasome, caspase activation, gasdermin D (GSDMD) cleavage‐mediated pore formation, and the release of inflammatory cytokines interleukin‐1 beta (IL1β) and interleukin‐18 (IL18), triggering an inflammatory response [13], and is one of the key mechanisms of the body’s inflammatory response. Previous studies have shown that ozone can activate the NF‐κB pathway in lung tissue, further promoting the activation of the NLRP3 inflammasome, ultimately leading to lung damage [14]. Another study demonstrated that ozone induces oxidative damage, generates reactive oxygen species (ROS), and activates mitochondrial autophagy, thereby activating the NLRP3 inflammasome [15]. The above results [14, 15] indicate that ozone can induce pyroptosis in lung tissue and lead to inflammation. Inflammation is a core pathological feature of asthma, with various inflammatory cells and mediators extensively involved in the persistent local inflammation of the airways. Prolonged inflammatory responses can cause permanent, irreversible changes in airway structure, including airway remodeling such as smooth muscle hyperplasia and thickening [16], and significantly increase airway reactivity [17]. Traditional Chinese medicine (TCM) is a unique treasure trove of medical knowledge in China. Salidroside is the primary component of the TCM Hongjingtian (*Rhodiola rosea*) and Hongjingtian injection (HJT). Studies have found that salidroside exerts multitarget inhibitory effects on the NLRP3/GSDMD pyroptosis pathway [18].

The inflammatory microenvironment of asthma comprises a complex network of diverse immune cells, structural cells, and cytokines. This microenvironment involves compromised airway epithelial barriers, matrix remodeling, and oxidative stress responses, collectively driving airway obstruction and chronic pathological changes. Ozone and irritant gases are significant triggers accelerating the development of the asthma inflammatory microenvironment. They induce various forms of cell death in airway epithelial cells, with pyroptosis being a key mechanism that exacerbates asthma progression. This study aims to analyze the role of pyroptosis in the exacerbation of asthma by ozone exposure using bioinformatics methods and asthma animal models. Furthermore, we will investigate the effects of targeting and regulating the pyroptosis pathway on airway inflammation and structural changes in ozone‐induced ovalbumin (OVA)‐sensitized asthma mice and explore the therapeutic potential of drugs in the pathogenesis of asthma induced by ozone.

## 2. Methods

### 2.1. Transcriptome Data Sources

In this study, asthma‐related transcriptome data (RNA‐seq) were collected from the Gene Expression Omnibus (GEO) database. The asthma data primarily included three tissue types: airway epithelial tissue, blood tissue, and nasal epithelial tissue. The airway epithelial tissue includes four cohorts: GSE63142, GSE43696, GSE67940, and GSE130499, comprising 405 asthma patients and 116 healthy controls. Blood tissue data were obtained from two cohorts: GSE69683 and GSE19301. GSE69683 consists of whole blood samples, including 87 healthy controls and 411 asthma patients. GSE19301 comprises peripheral blood mononuclear cells (PBMCs) from 685 asthma patients in the middle‐aged and elderly population. Nasal epithelial tissue was obtained from the GSE240567 cohort, which included 256 acute asthma patients and 297 healthy controls. Within this cohort, there were 234 middle‐aged and elderly individuals, including 107 acute asthma patients and 127 healthy controls. After obtaining the expression matrix, the data underwent normalization via FPKM conversion. Using the SVA package, we merged asthma tissue data and removed batch effects. The pyroptosis genes used in this study were obtained from the Molecular Signatures Database (MSigDB), which includes 27 genes.

### 2.2. Network Toxicology and Toxicological Analysis

Network toxicology is a new method specifically used to study the toxic components of drugs, employing bioinformatics and big data analysis methods to predict and evaluate the toxic mechanisms of drugs. This study utilized network toxicology to evaluate the toxic effects of ozone and its potential toxic targets. We collected ozone‐related toxicity genes from the comparative toxicogenomics database (CTD) [19], which contains 8281 genes. Among these, 16 genes are associated with pyroptosis. This study used the STRING database and Cytoscape to perform protein–protein interaction (PPI) analysis on the 16 genes, with the intensity of color indicating the core degree of the genes. Additionally, the toxicity effects of ozone were analyzed using the ADMETlab 3.0 database [20].

### 2.3. Functional Analysis and Immune Infiltration Analysis

This study performed functional analysis of pyroptosis‐related genes based on asthma airway epithelial tissue data. Pathway enrichment analysis was conducted on asthma data using 16 ozone‐related pyroptosis genes and GSVA analysis, yielding pathway enrichment scores (GSVA scores). Based on the pathway enrichment scores, asthma data were grouped into high‐score (high, score > 0) and low‐score (low, score < 0) groups, with 210 cases in the high‐score group and 195 cases in the low‐score group. Using limma analysis and GSEA enrichment analysis, we analyzed gene expression and functional differences between the two groups using the GO dataset and ReactomePA dataset [21]. The screening criteria for differentially expressed genes were logFC > 0.5 or logFC < −0.5, and *p* < 0.05.

This study used immune cell data from the mmc3 dataset [22] for immune infiltration analysis, which contains 28 immune cell‐related markers. We used ssGSEA analysis to analyze the immune cell infiltration levels in the airway epithelial tissue of asthma patients.

### 2.4. XGBoost Model and SHapley Additive exPlanations (SHAP) Analysis

XGBoost [23] uses gradient boosting to gradually construct a model. In the medical field, XGBoost excels at handling high‐dimensional, nonlinear omics data (such as gene expression profiles). It is frequently employed for complex tasks like disease diagnosis prediction and drug response classification, delivering highly interpretable results with stable performance. In this study, each dataset was randomly divided into training and testing sets in an 8:2 ratio. Based on 16 ozone‐related pyroptosis genes and asthma data from three types, a diagnostic prediction model was constructed.

SHAP analysis is a game theory‐based method for explaining machine learning model outputs [24]. In this study, SHAP analysis was used to analyze the role of ozone‐related pyroptosis genes in asthma diagnosis. Additionally, GAPDH was used as a reference gene, and the relative expression levels of ozone‐related pyroptosis genes were calculated based on the expression levels of GAPDH for model construction.

### 2.5. Mendelian Randomization (MR) Analysis of Drug Targets

The GWAS data used in this study were obtained from the MRC‐IEU, which included 53,598 asthma patients and 409,335 healthy controls. A total of 9,851,867 SNP loci were detected in this study, mainly from European populations. Based on asthma‐related GWAS data and SMR analysis, this study performed MR analysis on asthma. Two methods were employed in this study: SMR analysis and eQTL analysis. The eQTL analysis was conducted using GWAS data and eQTL data from 16 ozone‐related pyroptosis genes. The eQTL data from 16 ozone‐related pyroptosis genes were obtained from the eQTLGen Database. SMR analysis, short for summary‐data‐based MR, is a MR method based on summary data [25, 26]. It is primarily used for causal inference, particularly in studies related to gene expression. The reference coefficient used for SMR analysis is the *p*‐value (PSMR = 5 × 10^−6^), and the *p*‐value of the HEIDI test (PHEIDI > 0.05, indicating no significant heterogeneity). The STROBE‐MR checklist is shown in File S1.

### 2.6. Single‐Cell Data and Analysis

This study utilized single‐cell data (scRNA‐seq) from the GSE164015 cohort in the GEO database, which included alveolar lavage fluid from four placebo‐stimulated asthma patients and four allergen‐stimulated asthma patients. The dataset contained 49,104 cells. In this study, the Seurat package was used for quality control, data standardization, and batch effect removal. All cells were clustered and compared based on common bronchial cell markers. Furthermore, this study investigated the expression of 16 pyroptosis genes in different cell clusters in asthma pathological tissues and further analyzed the effect of allergen stimulation on gene expression.

To further characterize the role of GSDMD in the allergen‐stimulated asthma inflammatory microenvironment, this study classified two types of ciliated cells into GSDMD_positive and GSDMD_negative groups based on GSDMD expression. Furthermore, we performed AUCell pathway analysis on 10 inflammation‐related pathways and 18 cell death‐related pathways from the Hallmarker gene collection to assess the impact of GSDMD expression on the inflammatory microenvironment.

ScTenifoldKnk is an R‐based scRNA‐seq analysis package specifically designed for virtual gene knockout analysis. This tool employs mathematical modeling to simulate the impact of specific gene functional loss on gene regulatory networks, thereby predicting gene functions and regulatory relationships. This study further employed scTenifoldKnk to conduct virtual knockout analyses on GSDMD‐positive cell subpopulations, analyzing GSDMD’s influence on the inflammatory microenvironment by artificially suppressing its expression. We extracted 5448 inflammation‐associated genes from the GeneCards database and utilized virtual knockout to assess the impact of GSDMD suppression on the expression of these genes.

### 2.7. Asthma Animal Model and Validation

The classic mouse asthma model is an OVA‐induced airway inflammation model. The specific construction method is as follows: mice were sensitized with multiple intraperitoneal injections of OVA, followed by OVA challenge via aerosol inhalation to establish an acute asthma model (Figure S5A). In this model, serum IgE levels and eosinophil levels in bronchoalveolar lavage fluid increased, and histopathological staining of tissue sections showed increased airway mucus and inflammatory leukocyte infiltration. In this study, this animal model was used to establish four groups, each consisting of six mice. Zhidan Lu was aware of the group allocation at the different stages of the experiment. The groups included a control group (C0), an asthma group (A0), a low‐concentration ozone asthma group (ozone concentration 1.2 ppm, A1.2), and a high‐concentration ozone asthma group (ozone concentration 2.4 ppm, A2.4). Based on HE staining and α‐SMA staining, this study analyzed pathological changes in airway tissues between the control group and the three experimental groups. Using the Western blot experiment, the expression of cleaved‐GSDMD (cGSDMD), cleaved‐Casp1 (cCasp1), IL1β, and IL18 was detected in epithelial cell tissues of the four groups. Additionally, GAPDH was used as an internal reference gene. In this study, the animal model was established using sodium pentobarbital as the anesthetic, administered at a dose of 50 mg/kg via intraperitoneal injection. The mice were euthanized by cervical dislocation. This study has been approved by the Research Ethics Committee of the Second Hospital of Hebei Medical University (Number 2024‐AE402). The ARRIVE guidelines 2.0 is shown in File S2.

### 2.8. Network Pharmacology Analysis

The GSDMD gene is a core gene in the pyroptosis pathway. TCM is a traditional therapeutic method widely used in China. In this study, based on the Herb database and relevant literature, important TCM components related to GSDMD were screened to identify key herbal components related to GSDMD. This study screened drug targets associated with important components using SymMap, TCMID, TCMSP, TCM‐ID, and the Herb database. The STRING database and Cytoscape were used for PPI analysis to analyze the interactions among drug targets. Based on the SwissADME database, the drug‐development potential of relevant drugs was analyzed from five aspects: lipophilicity, molecular size, molecular polarity, insolubility, unsaturation, and molecular flexibility.

### 2.9. Molecular Clustering and Molecular Docking

This study used Discovery Studio to perform molecular clustering analysis, classifying the molecular sets of related TCM components based on their 3D structural features and extracting key molecules and their analogs for subsequent molecular docking. The structural data of the GSDMD protein were obtained from the PDB database, with a PDB ID of 721x and a resolution of 1.86 Å. All small molecule structural data were obtained from the PubChem database, with each molecule having a corresponding PubChem CID. Three methods were used for molecular docking in this study: Discovery Studio’s libDock, MOE’s molecular docking, and AutoDock’s molecular docking. This study considers docking results with libDockScore > 100 or binding energy < −5.5 kcal/mol to have significant research value. Moreover, the docking sites of each small molecule with the GSDMD protein are primarily located within a spherical region centered on amino acids 275–276 of the protein, with a radius of 5 Å. This is because this site is the primary site where GSDMD splits to produce the functional protein cGSDMD.

### 2.10. Drug Treatment Grouping and Disposal

A total of 42 male C57BL/6 wild‐type mice (SPF grade, 6–8 weeks old, weighing ~20 ± 2 g) were used in this experiment. All experimental mice underwent a 1‐week adaptive feeding period prior to the formal experiment. All animal experimental procedures were approved by the Research Ethics Committee of the Second Hospital of Hebei Medical University (Number 2024‐AE402). Zhidan Lu was aware of the group allocation at the different stages of the experiment. The mice were randomly divided into seven groups using a random number table, with six mice in each group: control + air + vehicle group (G1), asthma + air + vehicle group (G2), asthma + ozone + vehicle group (G3), asthma + ozone + MCC50 group (G4), asthma + ozone + VX765 group (G5), asthma + ozone + salidroside group (G6), and asthma + ozone + HJT group (G7).

### 2.11. Drug Administration Protocol

On days 15, 17, 19, 21, and 23 of the experiment, each group of experimental mice was administered the corresponding drug 30 min prior to each ozone exposure. All single doses were fully dissolved in a drug solution at a concentration of 10 μL/g based on the body weight of the experimental mice and administered via intraperitoneal injection, for a total of 5 days. The Vehicle group received a solution containing 5% DMSO, 40% PEG300, 5% Tween 80, and sterile injectable water in appropriate proportions, mixed according to the specified ratio. G1, G2, and G3 groups received 10 μL/g of vehicle per mouse per dose, G4 group received 10 μg/g of MCC950 per mouse per dose, G5 group received 30 μg/g of VX765 per mouse per dose, and G6 group received 50 μg/g of salidroside per mouse per dose, and Group G7 received 1.75 μL/g of HJT per mouse per dose (Figure 1A).

Figure 1Drug targets of Salidroside and Hongjingtian injection. (A) Analysis of the target functions of salidroside based on the GO gene set. (B) Analysis of the target functions of salidroside based on the ReactomePA pathway set. (C) Analysis of the target functions of HJT based on the GO gene set. (D) Analysis of the target functions of HJT based on the ReactomePA pathway set. (E) Association network of drug targets in HJT. (F) Association network between components of HJT and ozone‐related pyroptosis genes.

**Figure:**
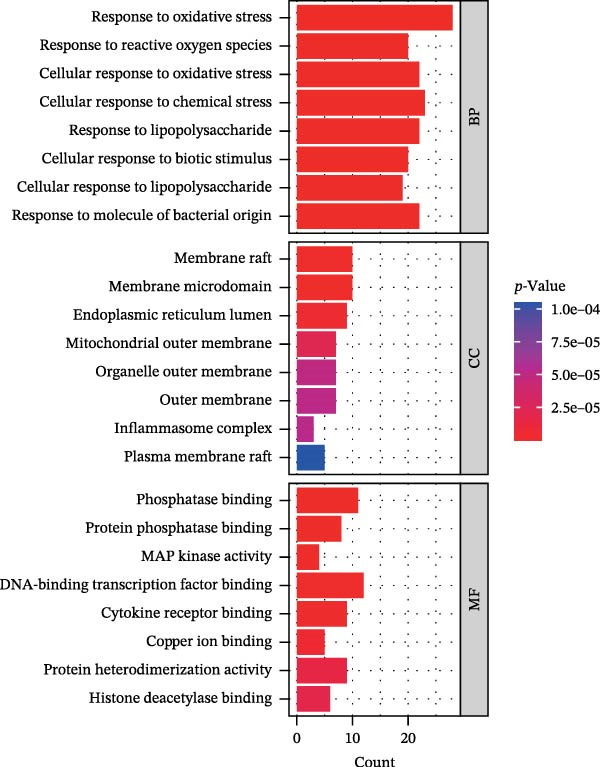
(A)

**Figure:**
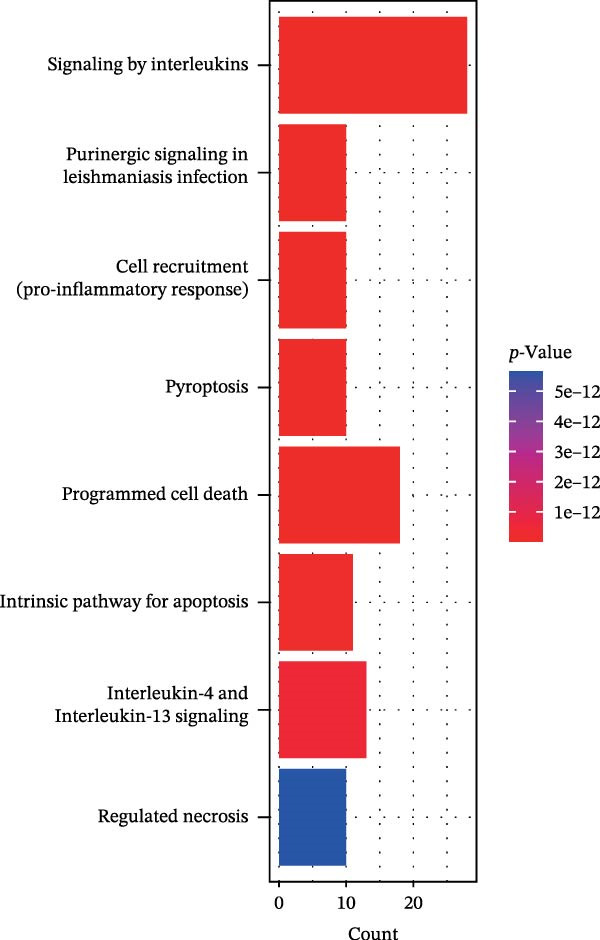
(B)

**Figure:**
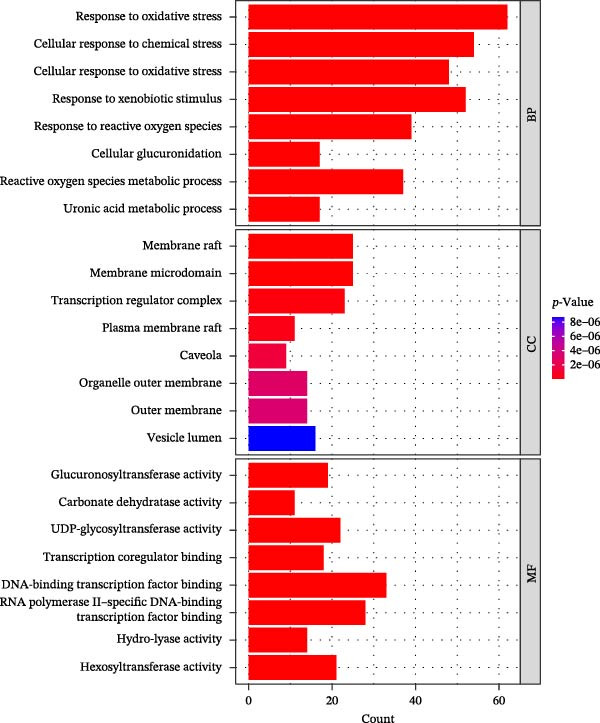
(C)

**Figure:**
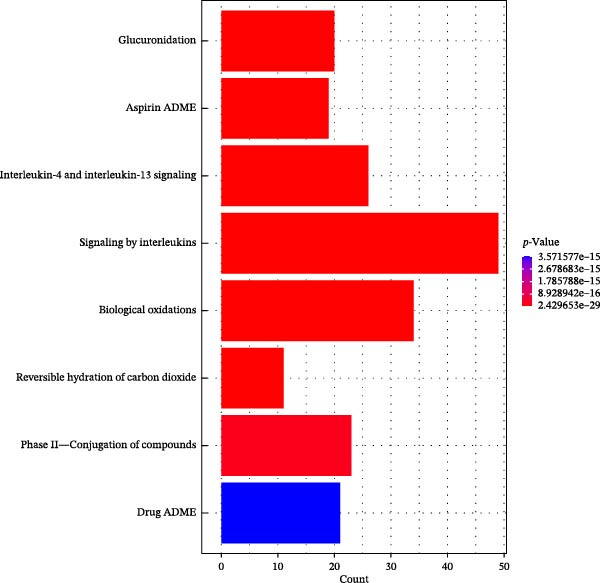
(D)

**Figure:**
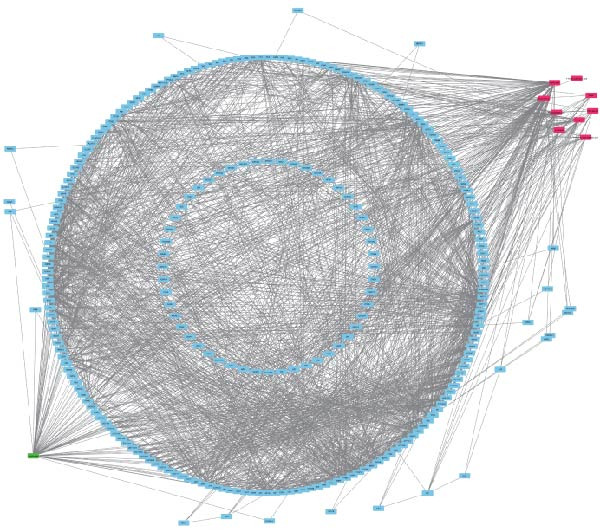
(E)

**Figure:**
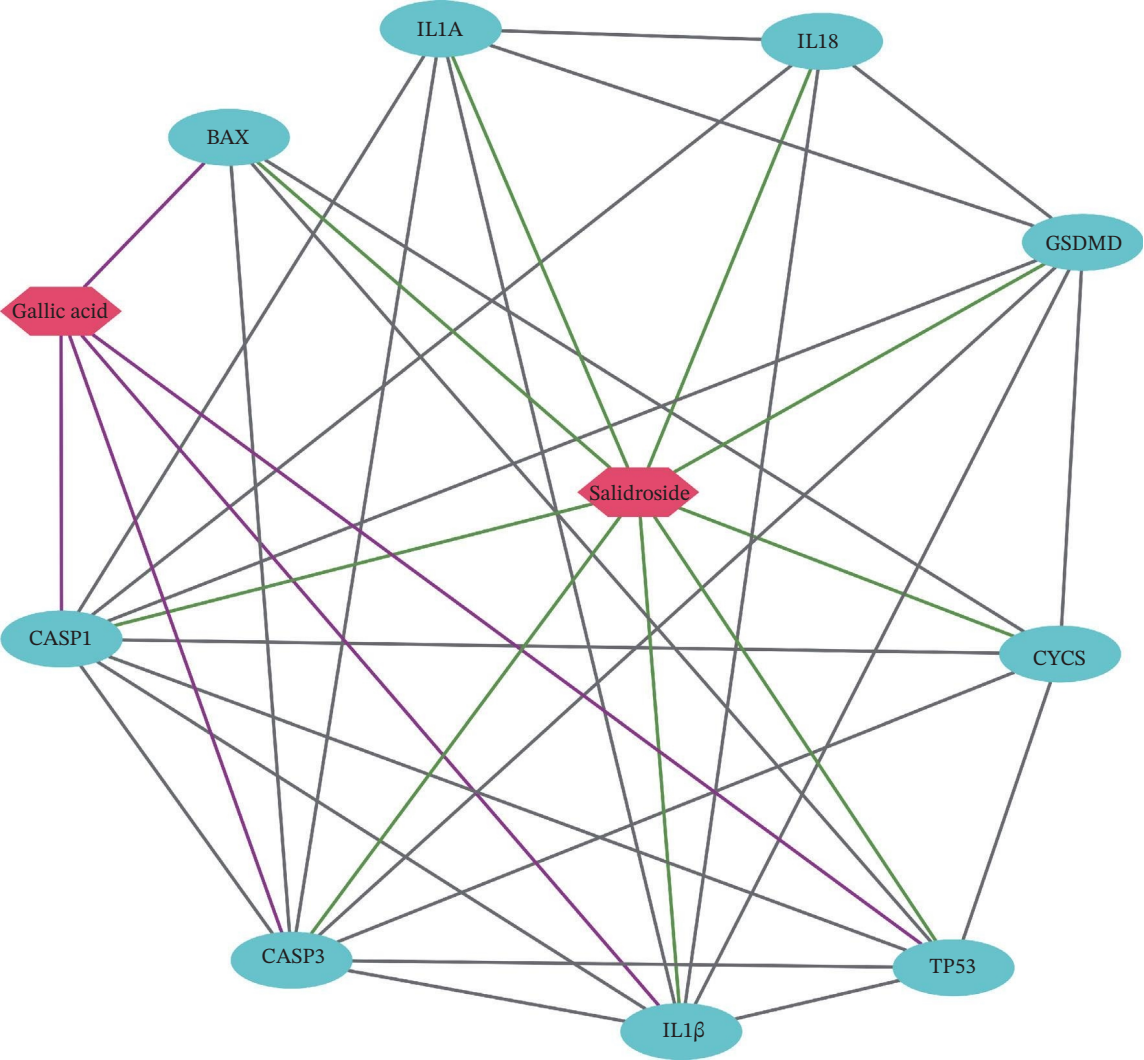
(F)

### 2.12. Detection After Drug Treatment

In this study, left lung tissue was extracted from the seven groups of mice after drug treatment, and tissue sections and protein samples were prepared. Based on HE staining and α‐SMA staining, pathological changes in the airway tissue of the seven groups of mice were analyzed. Based on Western blot analysis, the expression of cGSDMD, cleaved‐Casp1 (cCasp1), and NLRP3 in the epithelial cell tissues of the seven groups of animals was detected. Additionally, GAPDH was used as the internal reference gene. Based on immunofluorescence experiments, the expression distribution of GSDMD and NLRP3 in the lung tissues of the seven groups of mice was analyzed. Simultaneously, based on the in situ terminal deoxynucleotidyl transferase labeling technique (TUNEL), analyze the apoptosis status in the lung tissues of seven groups of mice. The ARRIVE guidelines 2.0 is shown in File S2.

### 2.13. Statistical Analysis

All experimental data are expressed as the mean ± standard error (SEM). Analysis was performed using SPSS (19.0), R studio (R4.2.1), Cytoscape (3.7.2), and Prism 9. Image J software was used for relative quantitative analysis of protein blot experiments and immunofluorescence experiments. Statistical analysis was performed using an independent samples *t*‐test to compare differences between groups. *p* value <0.05 was considered statistically significant.

## 3. Results

### 3.1. Expression Changes of Ozone‐Related Pyroptosis Genes in Asthma

Ozone is a common air pollutant and one of the major causative factors of respiratory‐related diseases. This study analyzed the toxic effects of ozone on the human body using the ADMETlab 3.0 database. Among the 20 toxic effects identified (Figure 2A), ozone may exhibit higher risks of drug‐induced liver injury (DILI), AMES toxicity, skin sensitization, carcinogenicity, eye corrosion, eye irritation, human hepatotoxicity, drug‐induced nephrotoxicity, hematotoxicity, and genotoxicity. This suggests that ozone poses significant health risks to humans.

Figure 2Screening of ozone‐related pyroptosis genes. (A) Prediction of ozone’s toxic effects. (B) Functions of ozone‐related genes based on the GO dataset. (C) Functions of ozone‐related genes based on the ReactomePA dataset. (D) PPI analysis of ozone‐related pyroptosis genes. (E) Expression of ozone‐related pyroptosis genes in asthma and normal conditions. (F) Expression of ozone‐related pyroptosis genes in severe asthma and nonsevere asthma.  ^∗^ *p* < 0.05;  ^∗∗^ *p* < 0.01;  ^∗∗∗^ *p* < 0.001;  ^∗∗∗∗^ *p* < 0.0001.

**Figure:**
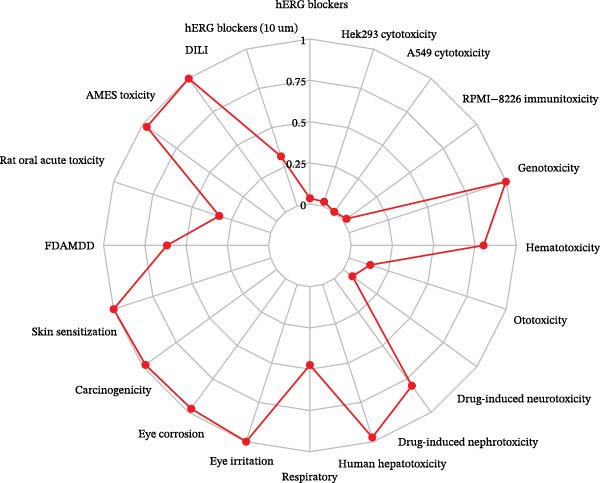
(A)

**Figure:**
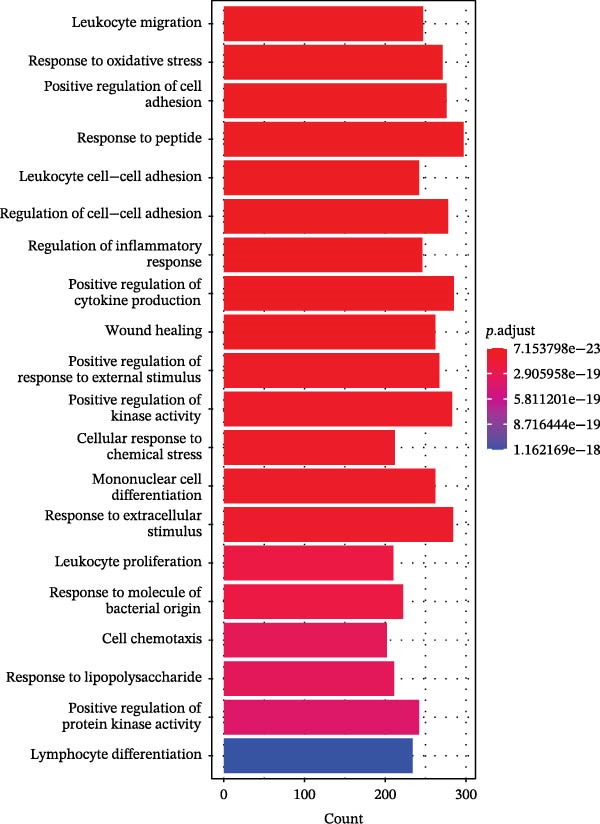
(B)

**Figure:**
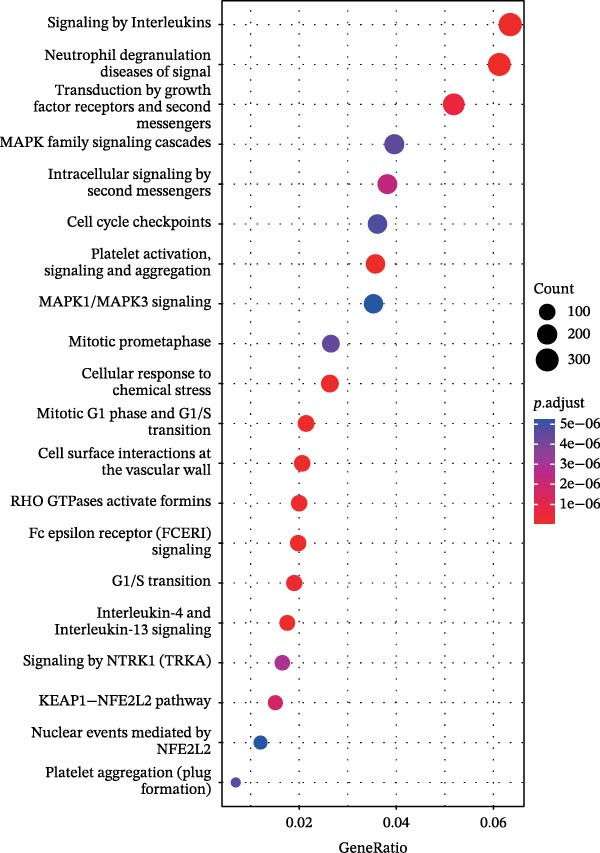
(C)

**Figure:**
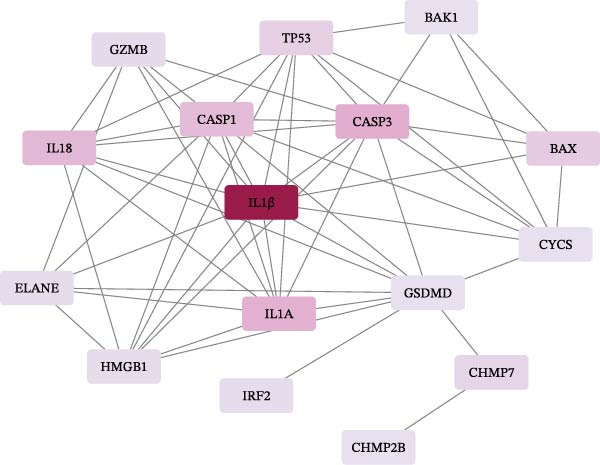
(D)

**Figure:**
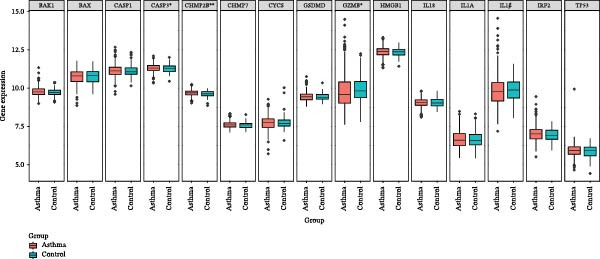
(E)

**Figure:**
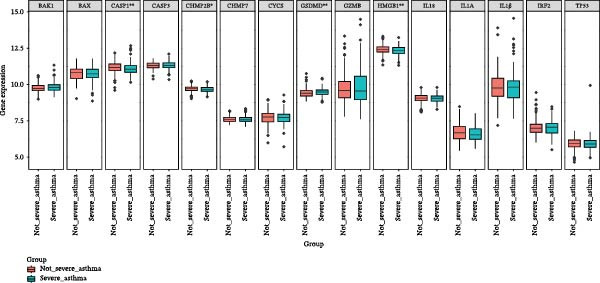
(F)

In this study, 8281 genes associated with ozone were identified from 18,369 studies in the CTD database. Through gene enrichment analysis based on the GO dataset (Figure 2B), we found that these genes are associated with leukocyte migration, response to oxidative stress, and positive regulation of cell adhesion. Based on the ReactomePA dataset (Figure 2C), we found that these genes are associated with signaling by interleukins, neutrophil degranulation, and interleukin‐4 and interleukin‐13 signaling. This suggests that ozone is associated with inflammation and interleukin‐related processes. We collected 27 cell pyroptosis‐related genes from the MSigDB database and found that 16 of them were associated with ozone (Figure 2D). These 16 genes are GSDMD, IL1β, CASP3, IL1A, CASP1, IL18, BAX, TP53, CHMP7, GZMB, ELANE, HMGB1, IRF2, BAK1, CHMP2B, and CYCS. PPI analysis identified IL1β as an important core gene, with IL1A, IL18, CASP1, CASP3, and BAX also playing significant roles.

The study collected RNA‐seq data from airway epithelial tissue of 405 asthma patients and 116 healthy controls, including 168 severe asthma patients and 237 nonsevere asthma patients. We found that the aforementioned 15 genes were expressed in the RNA‐seq data. In airway epithelial tissue, the expression levels of CASP3 and CHMP2B were higher in asthma patients than in healthy controls, while GZMB expression was reduced (Figure 2E). Additionally, compared to nonsevere asthma, severe asthma patients exhibited reduced expression of CASP1, CHMP2B, and HMGB1, while GSDMD expression was increased (Figure 2F).

Based on 15 ozone‐related pyroptosis genes and the GSVA algorithm, we calculated the enrichment level of the ozone‐related pyroptosis pathway in asthma patients. Compared with healthy individuals, asthma patients showed a higher enrichment level of the ozone‐related pyroptosis pathway (Figure S1A). Based on the GSVA scores of ozone‐related pyroptosis pathways in asthma patients, we divided them into a high‐score group (high, 210 cases) and a low‐score group (low, 195 cases) (Figure S1B). Based on RNA‐seq data from both groups and limma analysis (Figure S1C), we performed differential gene analysis, identifying 12 highly expressed genes and 9 lowly expressed genes. Furthermore, we found that the expression levels of GSDMD, IL1β, CASP3, IL1A, CASP1, IL18, BAX, TP53, CHMP7, GZMB, ELANE, IRF2, BAK1, CHMP2B, and CYCS were higher in the high group than in the low group (Figure S1D). GSEA enrichment analysis of the differentially expressed genes revealed that, based on the GO dataset, the high group exhibited increased enrichment of gene sets related to lymphocyte‐mediated immunity and decreased enrichment of gene sets related to axoneme (Figure S1E). Based on the ReactomePA dataset, we found that gene sets such as “Immunoregulatory interactions between a Lymphoid and a non‐Lymphoid cell” were enriched in the high group, while gene sets such as “Cell–cell junction organization” were enriched in the low group (Figure S1F). In this study, we performed infiltration analysis of 28 immune cell types using the ssGSEA method. The results showed that the infiltration levels of 24 immune cells were higher in the high group than in the low group, with activated CD4 T cell, activated CD8 T cell, and activated dendritic cells showing significant increases in infiltration (Figure 3A). This suggests that the enhancement of the ozone‐related pyroptosis pathway in asthma promotes the enhancement of immune responses in airway epithelial tissue.

Figure 3Diagnostic role of ozone‐related pyroptosis genes in airway epithelial tissue. (A) Effects of ozone‐related pyroptosis genes on immune cell infiltration. (B) ROC curve of the training set for the asthma disease prediction model. (C) ROC curve of the test set for the asthma disease prediction model. (D) Distribution of SHAP values for the asthma disease prediction model. (E) Average SHAP values of the asthma disease prediction model. (F) ROC curve of the training set for the severe asthma prediction model. (G) ROC curve of the test set for the severe asthma prediction model. (H) Distribution of SHAP values for the severe asthma prediction model. (I) Average SHAP value of the severe asthma prediction model.  ^∗^ *p* < 0.05;  ^∗∗^ *p* < 0.01;  ^∗∗∗^ *p* < 0.001;  ^∗∗∗∗^ *p*  < 0.0001.

**Figure:**
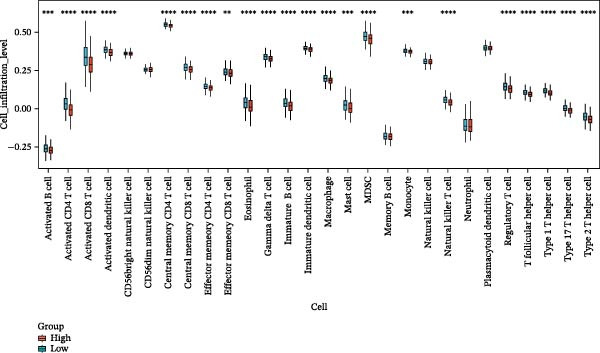
(A)

**Figure:**
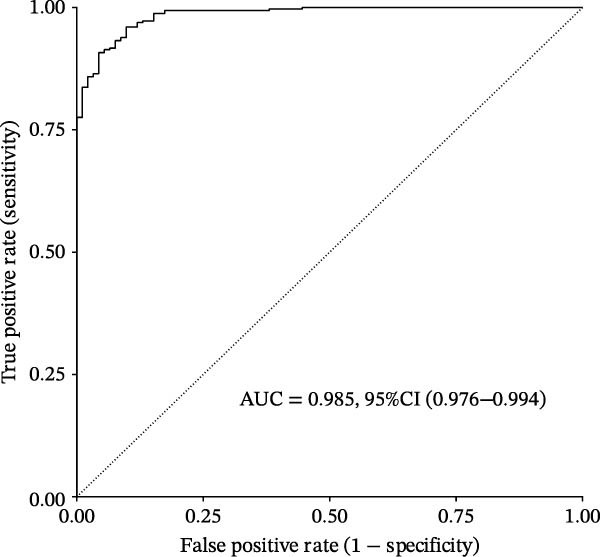
(B)

**Figure:**
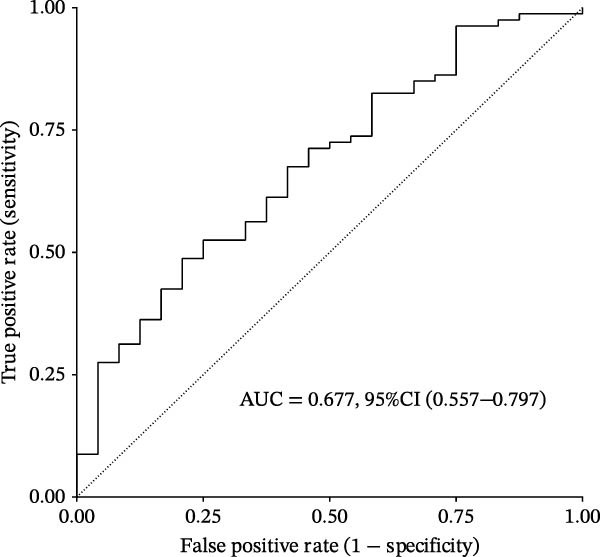
(C)

**Figure:**
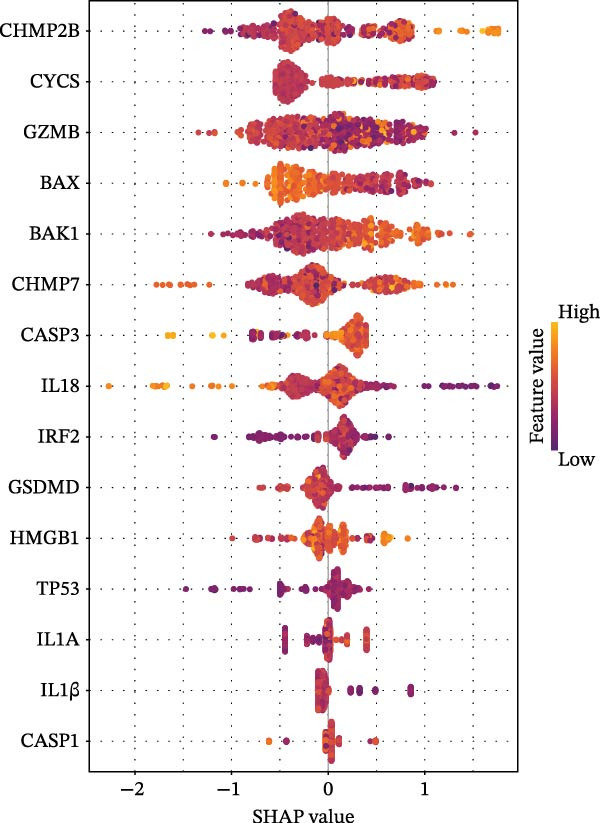
(D)

**Figure:**
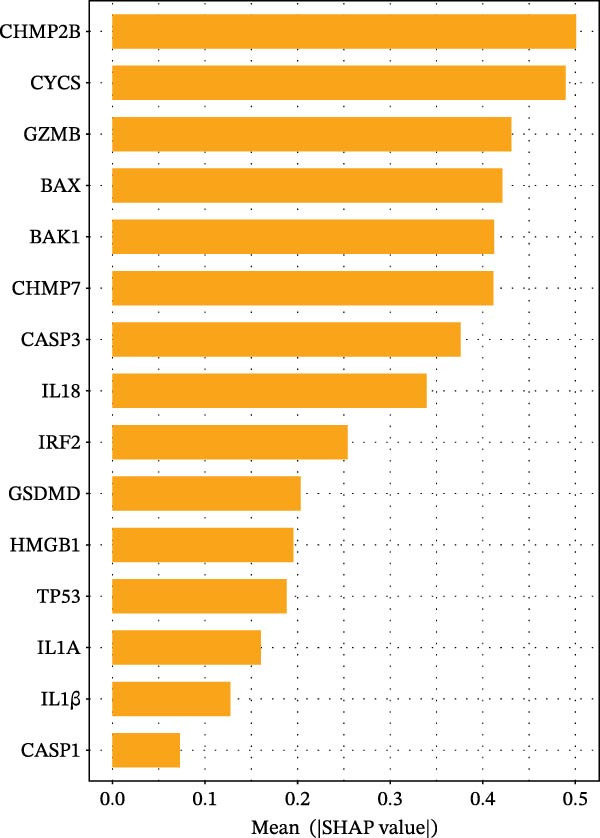
(E)

**Figure:**
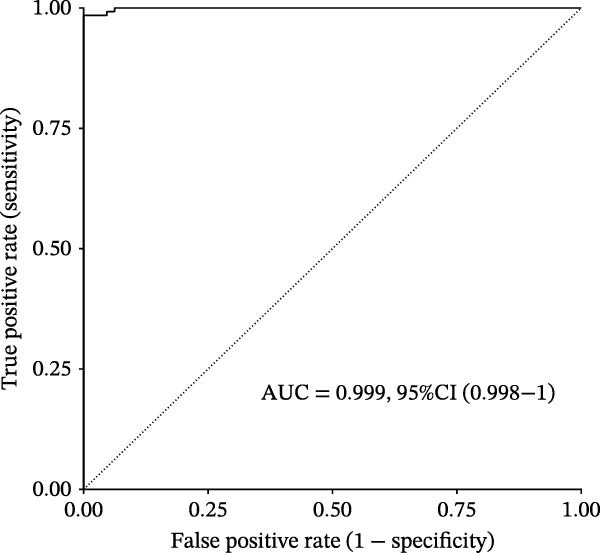
(F)

**Figure:**
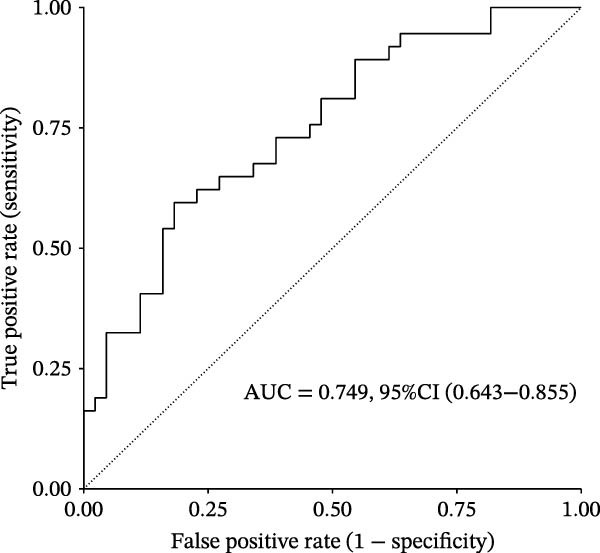
(G)

**Figure:**
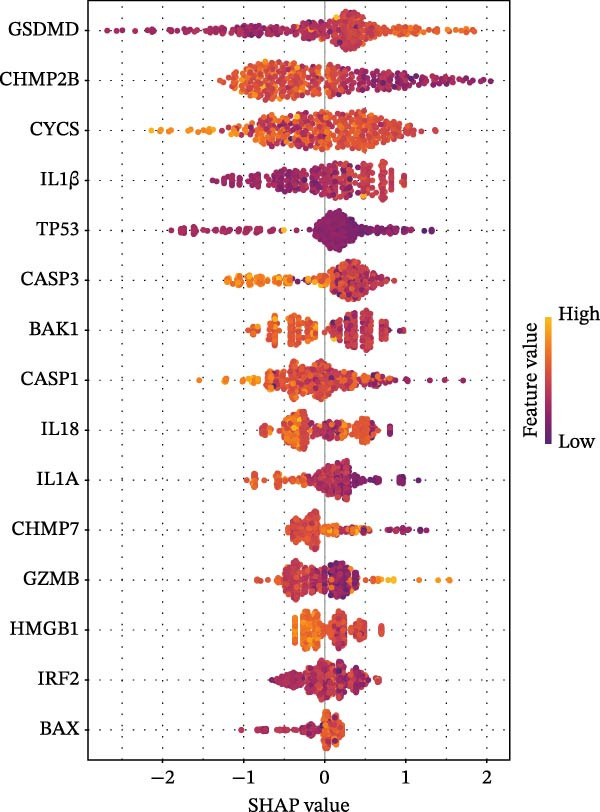
(H)

**Figure:**
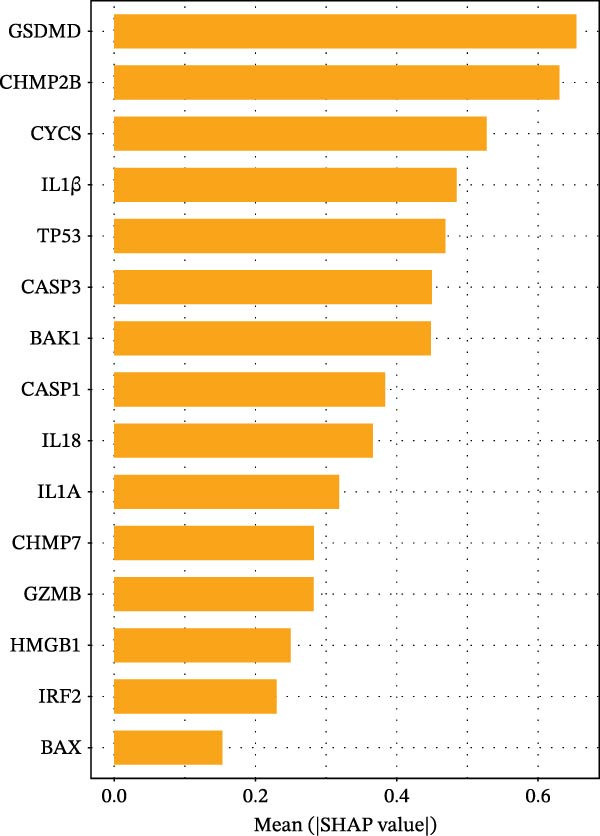
(I)

### 3.2. Diagnostic Predictive Role of Ozone‐Related Pyroptosis Genes in Asthma Patients

Based on XGBoost and SHAP analysis, we can use ozone‐related pyroptosis genes as feature variables to predict important outcomes in asthma disease progression. This study analyzed the diagnostic role of ozone‐related pyroptosis genes in the clinical progression of asthma based on gene expression data from three types of samples: airway epithelial tissue, blood tissue, and nasal epithelial tissue. This study collected 521 airway epithelial tissue data, including 405 asthma patients and 116 healthy controls.

Among the 405 asthma patients, 237 had nonsevere asthma and 168 had severe asthma. The gene expression data included expression data for 15 ozone‐related pyroptosis genes. Based on the XGBoost method and gene expression data, we constructed an asthma disease prediction model to predict whether patients have asthma. The ROC curve shows that the AUC value of the training set is 0.985 (0.976–0.994) (Figure 3B). A random 20% of the data was used as the test set for validation, showing an AUC value of 0.677 (0.577–0.797) (Figure 3C). SHAP analysis revealed that GZMB and CHMP2B exhibit strong diagnostic predictive roles (Figure 3E). Among these, GZMB shows a significant increase in SHAP values with rising gene expression (Figure 3D), indicating it is an important risk factor for asthma development. In this study, we also constructed a severe asthma prediction model to predict whether asthma patients have severe asthma. The ROC curve shows that the AUC value of the training set is 0.999 (0.9998–1) (Figure 3F). A random 20% of the data was used as the test set for validation, yielding an AUC value of 0.749 (0.643–0.855) (Figure 3G). SHAP analysis revealed that GSDMD and IL1β exhibit strong diagnostic predictive roles (Figure 3I). Additionally, as gene expression increases, SHAP values show a significant upward trend (Figure 3H), indicating that these genes are important risk factors for severe asthma.

The results of predictive models based on other asthma‐related tissue data, including blood, nasal epithelium, and PBMCs, can be found in File S3.

In summary, from the aforementioned asthma‐related tissue cohorts, it can be observed that ozone‐related pyroptosis genes exhibit strong diagnostic predictive roles in asthma disease progression. Furthermore, GSDMD, IL1β, IL18, and CASP1 play more important roles in asthma exacerbation and progression.

### 3.3. Ozone Exacerbates Asthma by Activating the Cell Pyroptosis Pathway

In this study, we used an OVA‐induced asthma animal model (Figure S5A) to detect the expression of key pyroptosis genes. Four groups were established (Figure 4A), with six mice in each group. These groups included the C0 group, A0 group, A1.2 group, and A2.4 group. This study detected the expression of cGSDMD, cleaved‐Casp1 (cCasp1), IL1β, and IL18 in the epithelial tissue of the four groups of animals to reflect the activation of the pyroptosis pathway in asthma patients under ozone exposure. The results showed that with increasing ozone concentration, the epithelial tissues of asthma mice exhibited high expression of cGSDMD, cCasp1, IL1β, and IL18 (Figure 4A). Furthermore, Image J and grayscale analysis revealed that with increasing ozone concentration, the expression of all four genes significantly increased, with statistical significance (Figure S5C).

Figure 4Experimental validation of asthma models and drug target analysis. (A) Expression of key pyroptosis‐related genes in asthma animal models. (B) Changes in airway epithelial tissue in asthma animal models. 1, C0 group; 2, A0 group; 3, A1.2 group; 4, A2.4 group. (C) Target genes associated with asthma. (D) SNP loci associated with GSDMD. (E) Target genes associated with the rs4874140 locus.

**Figure:**
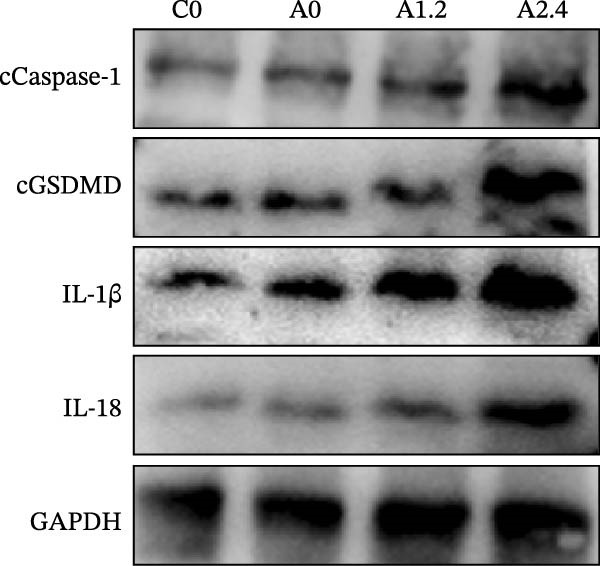
(A)

**Figure:**
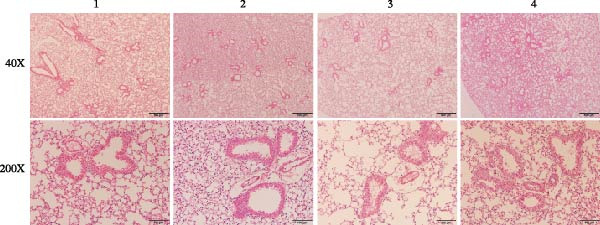
(B)

**Figure:**
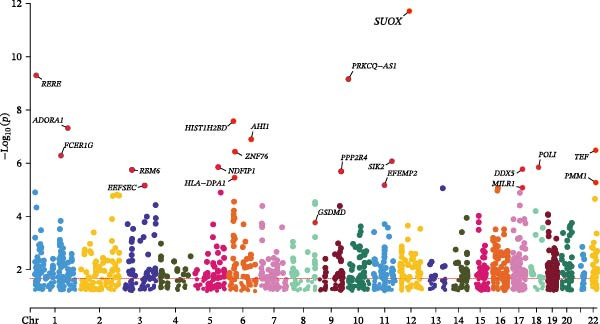
(C)

**Figure:**
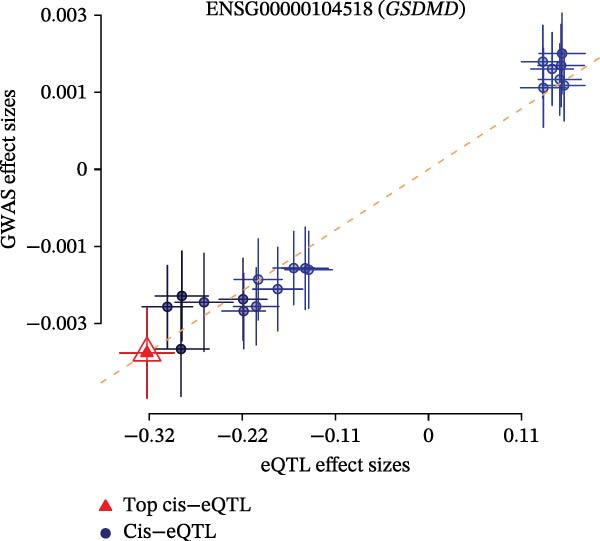
(D)

**Figure:**
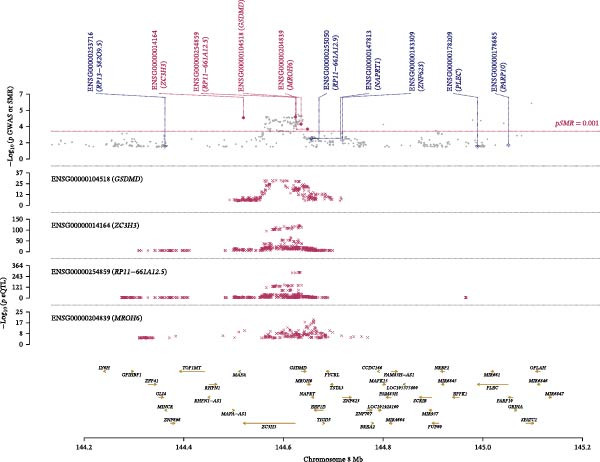
(E)

In this study, HE staining was used to stain the airway epithelial tissue of the control group and three experimental groups. The results (Figure 4B) showed that compared with the C0 group, the three asthma groups exhibited local infiltration of inflammatory cells, which increased with increasing ozone concentration. In this study, α‐SMA immunohistochemical staining was used to assess changes in airway structure in experimental mice. The results (Figure S5B) showed that compared with the C0 group, α‐SMA expression significantly increased in the airway surrounding area in the three asthma groups, and this increase was further enhanced with increasing ozone exposure concentration. This finding indicates that exposure to different concentrations of ozone exacerbates airway smooth muscle hyperplasia and hypertrophy, thereby worsening airway structural changes in OVA‐induced asthma mice.

### 3.4. Multomics Analysis of Ozone‐Related Pyroptosis Key Genes in Asthma

Based on asthma GWAS data and SMR analysis methods, we analyzed asthma‐related target genes and identified 754 target genes (Figure 4C). Among these, GSDMD is the target gene significantly associated with asthma among ozone‐related pyroptosis genes, with SMR detection showing *p* = 0.000171 and HEIDI detection *p* = 0.828. In asthma data, the SNP significantly associated with GSDMD (top‐cis eQTL) is rs4874140 (Figure 4D). This locus is located at chromosome 8 at position 144571841, with a mutation rate of 0.1948, and is associated with multiple genes (GSDMD, ZC3H3, and MROH6) (Figure 4E). Additionally, based on eQTL analysis, this study found that GSDMD promotes asthma onset (Figure S5D) and is associated with three SNP loci (Figure S5E). In summary, based on drug target MR analysis results, GSDMD may be an important target gene in asthma patients and could serve as a potential therapeutic target for asthma.

Ozone is a common irritant gas, and elevated concentrations act as an allergen that exacerbates asthma. In this study, we analyzed the expression of cell pyroptosis‐related genes in various cell populations of asthma pathological tissues using a scRNA‐seq cohort exposed to allergen stimulation. Using common marker genes of bronchial epithelium, we classified the 49,104 cells in the scRNA‐seq cohort into nine cell populations (Figure S6A). Among these, bronchial epithelial cells include ciliated cells, goblet cells, and stromal cells, with ciliated cells further divided into functional cells and mitotic cells. By comparing the distribution of cell clusters between allergen‐stimulated asthma samples (AC group) and the control group, we found an increase in functional ciliated cells and goblet cells in bronchoalveolar lavage fluid after allergen stimulation (Figure S6B). Furthermore, compared with the control group, GSDMD and IL18 were significantly overexpressed in both cell types after allergen stimulation (Figure S6C,D). Additionally, 16 ozone‐related pyroptosis‐related genes showed varying degrees of increased expression in bronchial epithelial cells after allergen stimulation (Figure S6E). This suggests that allergen stimulation promotes pyroptosis and exacerbates asthma progression.

### 3.5. Impact of GSDMD on the Asthma Microenvironment Based on Virtual Knockout Analysis

Asthma is a disease caused by chronic inflammation, and allergens such as ozone can exacerbate its progression by promoting inflammation in airway tissues. scRNA‐seq analysis revealed that two ciliated cell subpopulations (functional ciliated and mitotic ciliated) exhibited elevated GSDMD expression upon allergen stimulation (Figure 5A). This study classified the functional ciliated subpopulation into GSDMD_positive and GSDMD_negative group based on GSDMD expression (Figure 5B). The GSDMD_positive group exhibited a greater susceptibility to multiple forms of cell death compared to the GSDMD_negative group (Figure 5C). Furthermore, the GSDMD_positive group demonstrated increased activation of inflammation‐related pathways (Figure 5D). Similarly, the mitotic ciliated subpopulation was divided into GSDMD_positive and GSDMD_negative group (Figure 5E). Results within the GSDMD_positive group mirrored those observed in the functional ciliated subpopulation (Figure 5F,G).

Figure 5Effects of GSDMD expression on the asthma inflammatory microenvironment. (A) GSDMD expression in asthma cell subpopulations. (B) GSDMD‐associated grouping within the functional ciliated. (C) Impact of GSDMD expression on cell death within the functional ciliated. (D) Effect of GSDMD expression on inflammatory pathways in the functional ciliated. (E) GSDMD‐associated clustering in the mitotic ciliated. (F) Effect of GSDMD expression on cell death in the mitotic ciliated. (G) Effect of GSDMD expression on inflammatory pathways in the mitotic ciliated.

**Figure:**
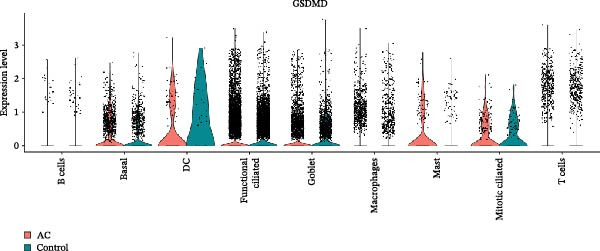
(A)

**Figure:**
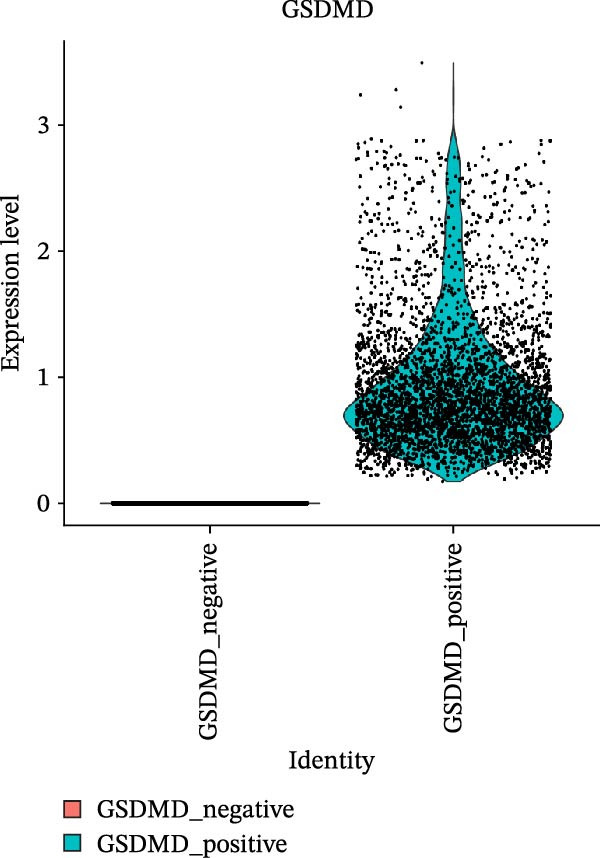
(B)

**Figure:**
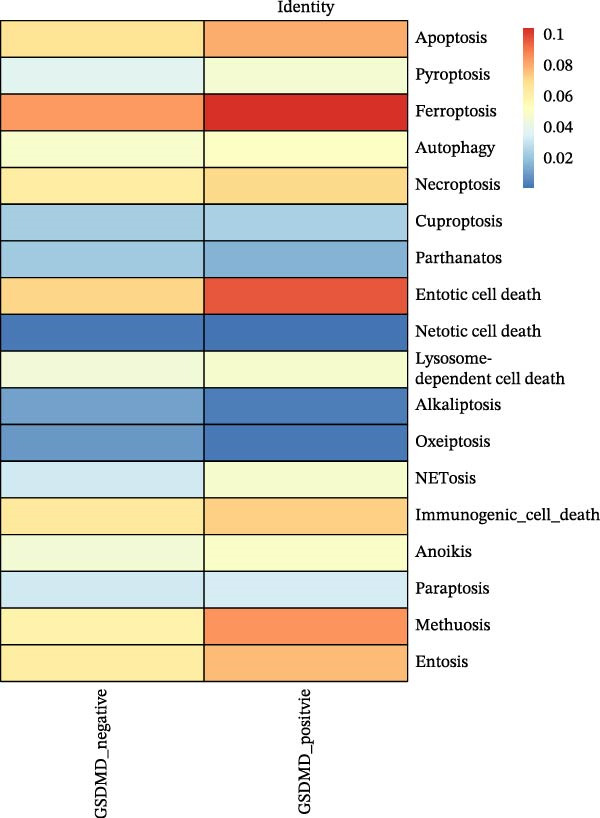
(C)

**Figure:**
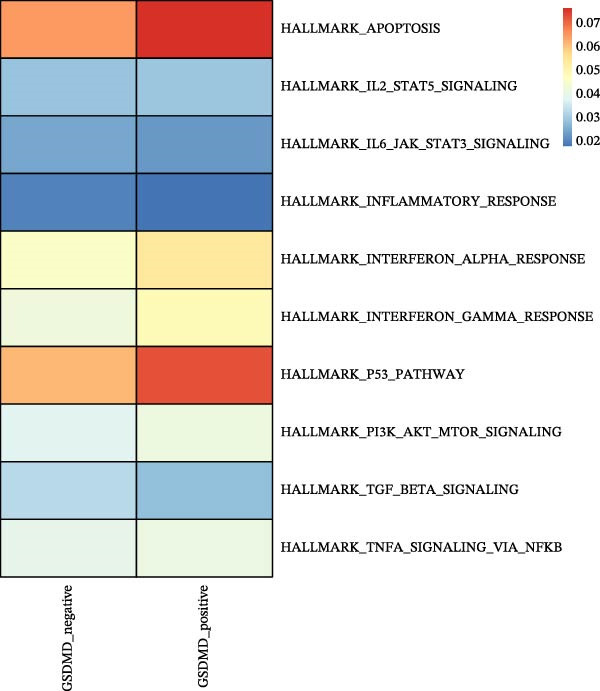
(D)

**Figure:**
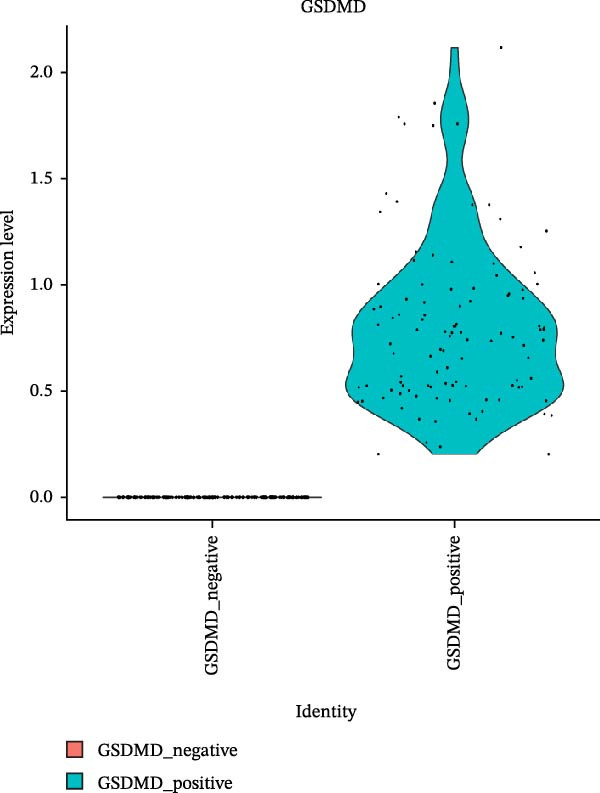
(E)

**Figure:**
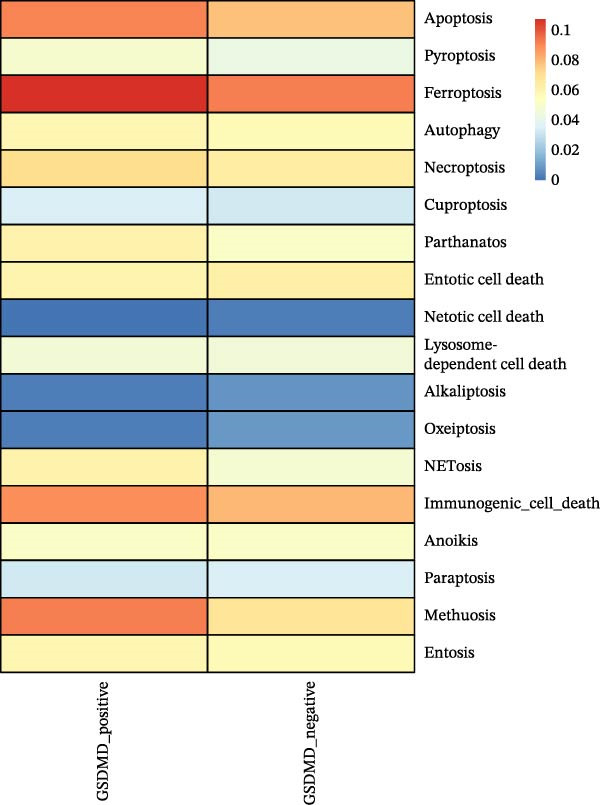
(F)

**Figure:**
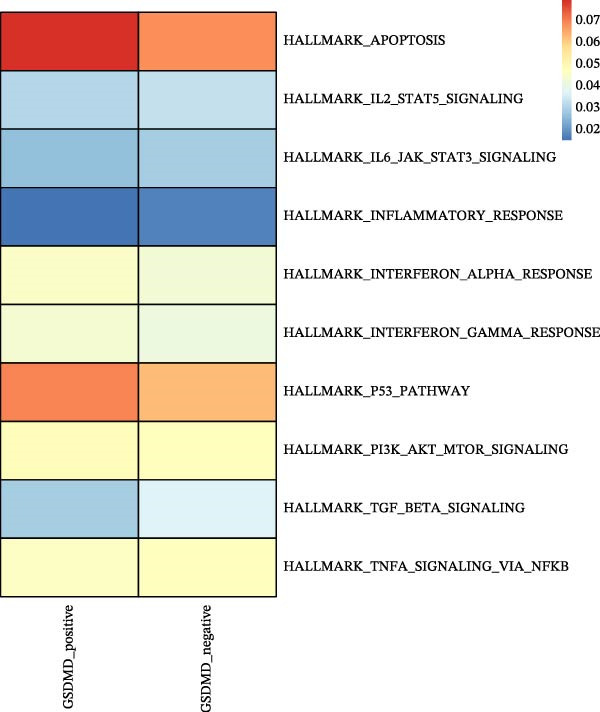
(G)

To further investigate GSDMD’s influence on the inflammatory microenvironment, we performed virtual knockout of GSDMD in the GSDMD_positive group of both subpopulations, analyzing changes in inflammation‐related genes following GSDMD removal. Results revealed 3348 genes exhibiting altered expression in the functional ciliated post‐knockout. Specifically, 375 genes showed increased expression (Figure 6A), while 2973 genes exhibited decreased expression (Figure 6D). GO enrichment analysis revealed that upregulated genes were predominantly associated with ciliary cell motility (Figure 6B), while downregulated genes were primarily linked to inflammatory responses (Figure 6E). KEGG enrichment analysis indicated that upregulated genes were mainly associated with motor proteins (Figure 6C), whereas downregulated genes were predominantly related to inflammatory pathways (Figure 6F). Similarly, GSDMD knockout in the mitotic ciliated resulted in altered expression of 4248 genes. Specifically, 740 genes exhibited increased expression (Figure 6G), while 3708 genes showed decreased expression (Figure 6J). GO enrichment analysis indicated that upregulated genes were predominantly associated with ciliated cell movement (Figure 6H), whereas downregulated genes were primarily linked to inflammatory responses (Figure 6K). KEGG enrichment analysis indicated that the upregulated genes were predominantly associated with motor proteins (Figure 6I), whereas the downregulated genes were primarily linked to inflammatory pathways (Figure 6L). In summary, GSDMD knockout promotes functional recovery in both types of ciliated cells and suppresses the occurrence of immune inflammatory responses.

Figure 6Effects of virtual GSDMD knockout on inflammation‐associated genes. (A) Top 20 genes elevated following GSDMD knockout in the functional ciliated. (B) GO analysis of genes elevated after GSDMD knockout in the functional ciliated. (C) KEGG analysis of genes elevated after GSDMD knockout in the functional ciliated. (D) Top 20 downregulated genes following GSDMD knockout in the functional ciliated. (E) GO analysis of downregulated genes after GSDMD knockout in the functional ciliated. (F) KEGG analysis of downregulated genes after GSDMD knockout in the functional ciliated. (G) Top 20 genes upregulated following GSDMD knockout in the mitotic ciliated. (H) GO analysis of genes upregulated following GSDMD knockout in the mitotic ciliated. (I) KEGG analysis of genes upregulated following GSDMD knockout in the mitotic ciliated. (J) Top 20 downregulated genes following GSDMD knockout in the mitotic ciliated. (K) GO analysis of downregulated genes following GSDMD knockout in the mitotic ciliated. (L) KEGG analysis of downregulated genes following GSDMD knockout in the mitotic ciliated.

**Figure:**
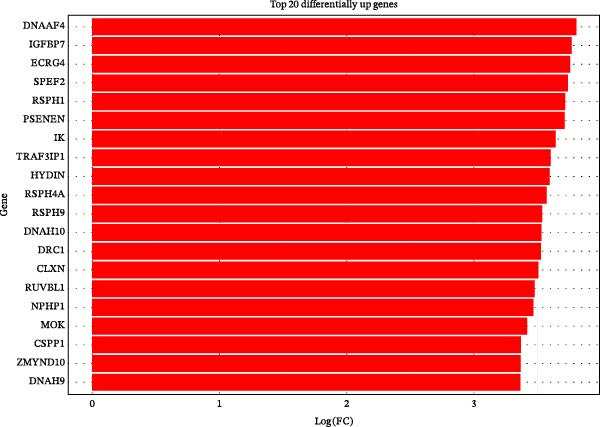
(A)

**Figure:**
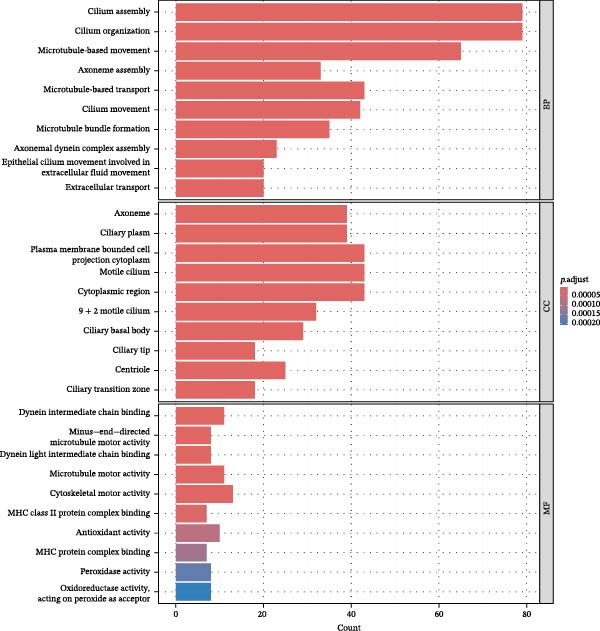
(B)

**Figure:**
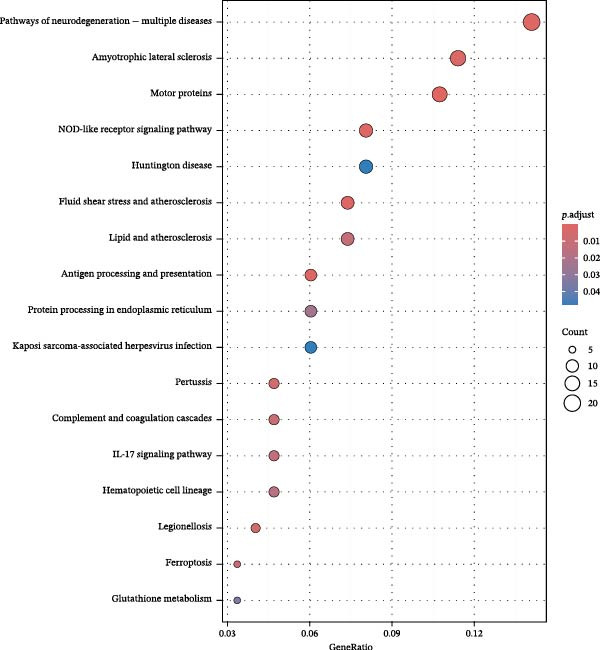
(C)

**Figure:**
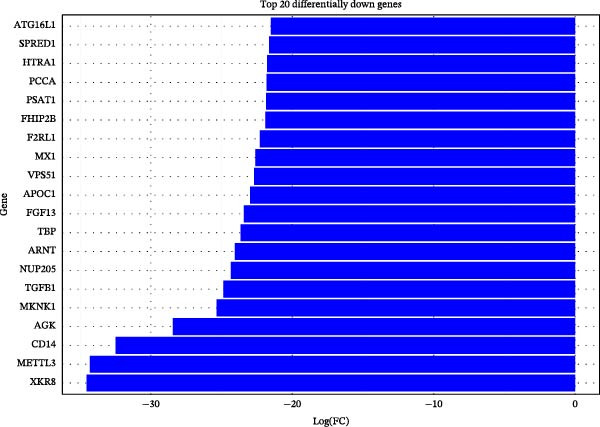
(D)

**Figure:**
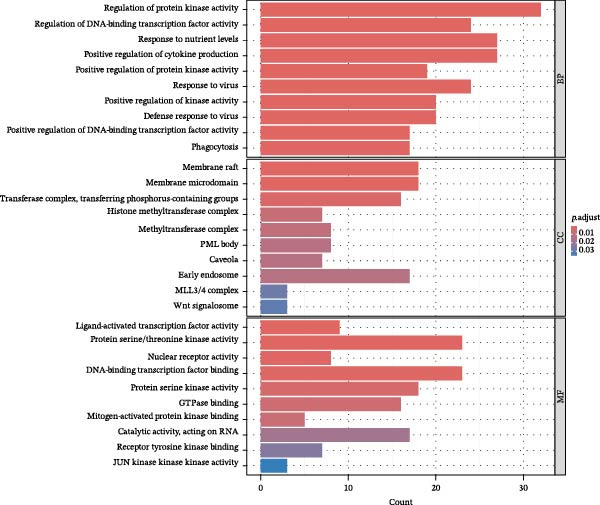
(E)

**Figure:**
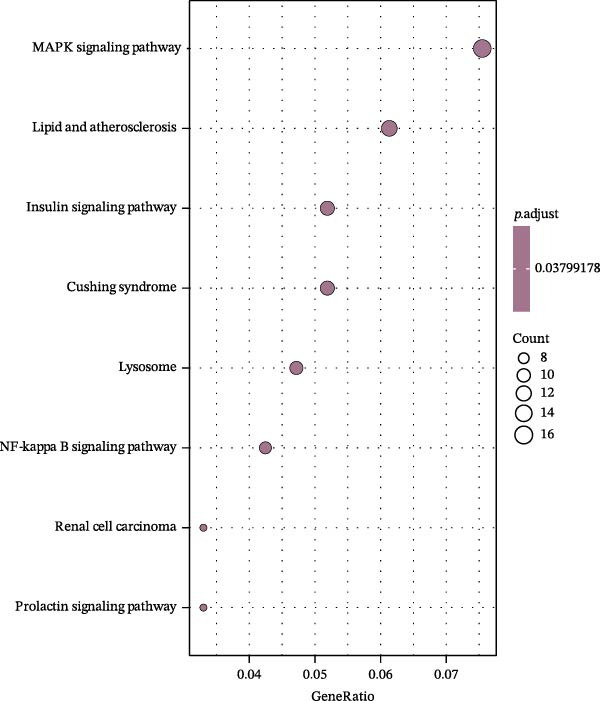
(F)

**Figure:**
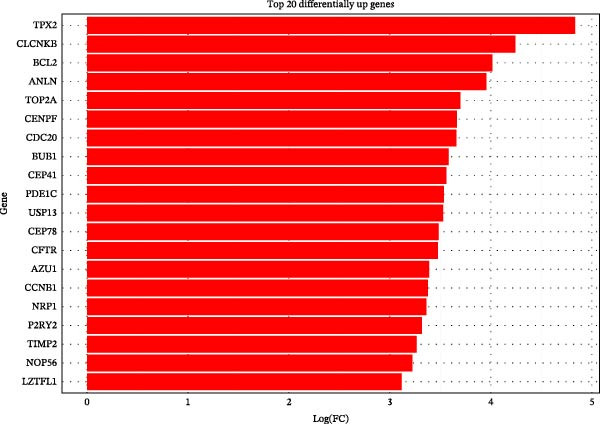
(G)

**Figure:**
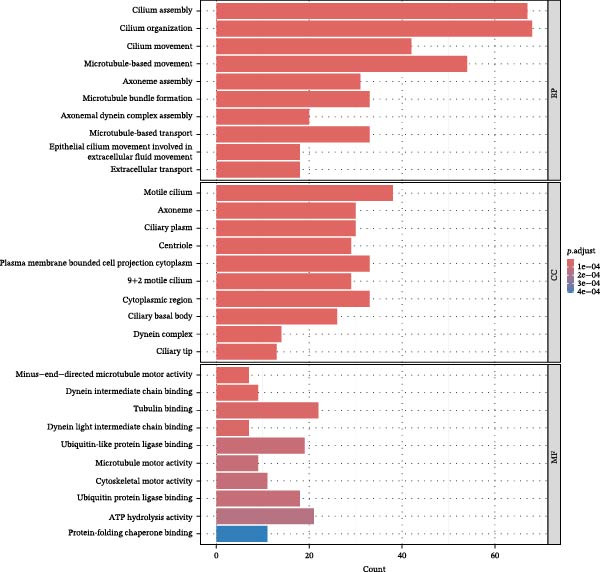
(H)

**Figure:**
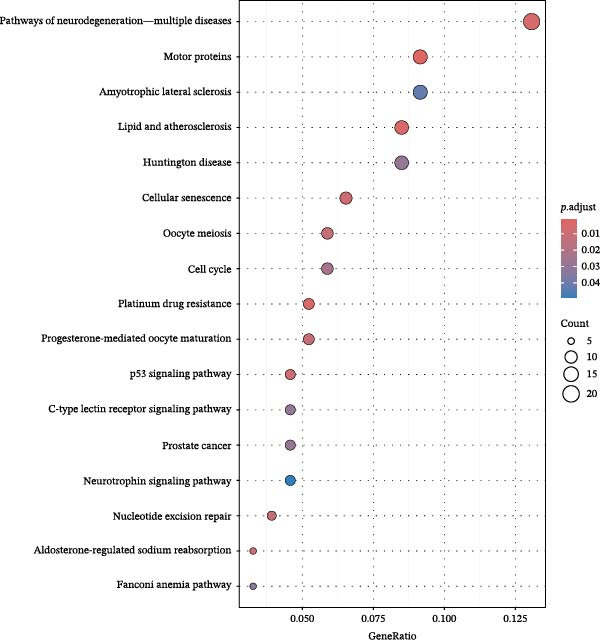
(I)

**Figure:**
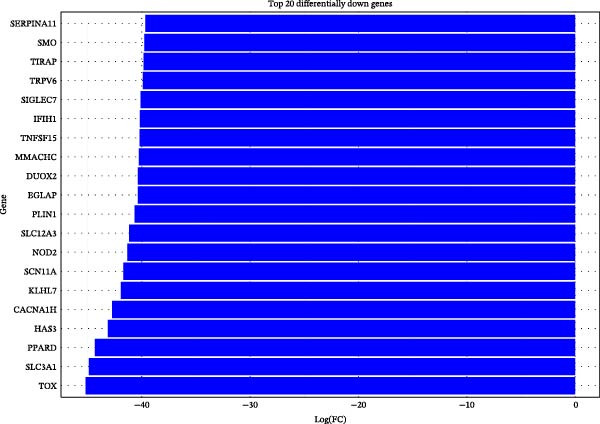
(J)

**Figure:**
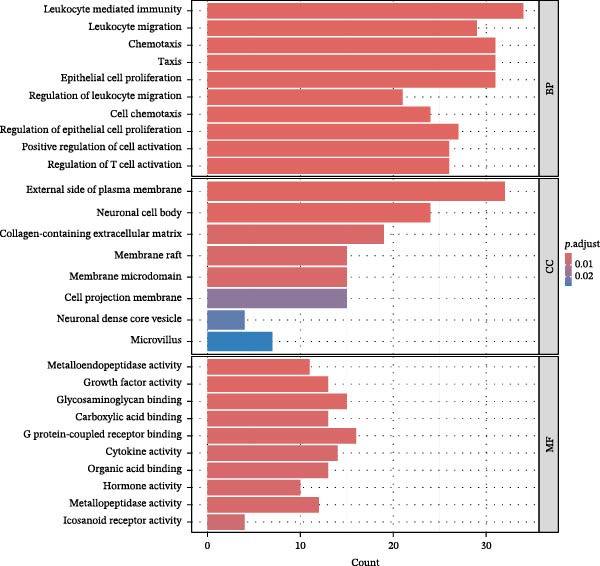
(K)

**Figure:**
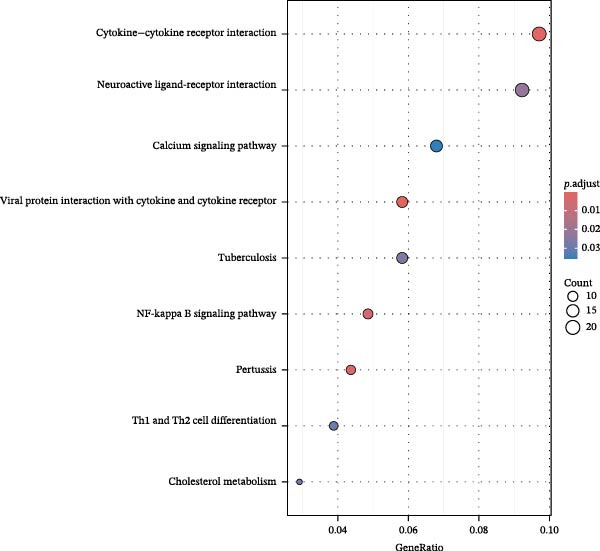
(L)

### 3.6. Network Pharmacology Analysis and Screening of Drugs Related to Pyroptosis Key Genes

GSDMD is a core gene involved in cell pyroptosis, and multiomics analysis has revealed that GSDMD plays a role in the onset and progression of asthma. In this study, based on the Herb database and relevant literature, we screened important TCM components related to GSDMD and identified four key TCM components associated with GSDMD (Table S1, supported by at least two references). Using the SwissADME database, we analyzed the drug‐like properties of the four TCM components from five aspects: lipophilicity, molecular size, molecular polarity, insolubility, unsaturation, and molecular flexibility. Among them, emodin and wedelolactone have poor unsaturation (Figure S7A,B). Melatonin and salidroside exhibit good drug‐like properties (Figure S7C,D), but melatonin, as a common hormone, regulates circadian rhythms and is unsuitable for asthma treatment. Therefore, this study selected salidroside as the primary research drug.

In this study, we screened salidroside’s targets using SymMap, TCMID, TCMSP, TCM‐ID, and Herb databases, identifying 81 targets (Table S3). Based on the GO gene set, enrichment analysis showed that the 81 targets were primarily associated with environmental stress (Figure 1A).Based on the ReactomePA pathway gene set, enrichment analysis showed that the 81 targets were primarily associated with the interleukin pathway and the pyroptosis pathway (Figure 1B). Salidroside is currently not used in clinical treatment, and HJT is a common therapeutic drug containing salidroside. Related literature [27] has identified the main components of HJT. In this study, based on Lipinski’s principle, the main components of HJT were screened, and 17 bioactive components containing salidroside were identified (Table S2). In this study, we screened the targets of the above components using SymMap, TCMID, TCMSP, TCM‐ID, and Herb databases, identifying 264 targets (Table S3). PPI analysis showed that these targets have strong associations (Figure 1E). Based on the Go gene set, enrichment analysis showed that the 264 targets are also mainly related to environmental stress (Figure 1C). Based on ReactomePA pathway gene sets, enrichment analysis showed that the 264 targets were mainly associated with the interleukin pathway and drug metabolism (Figure 1D). Further analysis revealed that, based on current database data, only salidroside and Gallic acid were associated with 9 genes of 16 pyroptosis genes (Figure 1F). This suggests that salidroside is a compound with potential research value.

### 3.7. Molecular Docking Validation of Salidroside and Its Analogs

By examining the 17 key components of HJT, we identified components with structures similar to salidroside, and most of these components lack corresponding targets in the TCM database. Based on 3D structures, this study performed molecular clustering analysis of the 17 components using Discovery Studio (Figure 7A), identifying seven components structurally similar to salidroside. The seven components are 4‐(beta‐D‐Glucopyranosyloxy)‐3‐ methoxybenzoic acid (PubChem CID: 14132337), [3,4,5‐trihydroxy‐6‐(hydroxymethyl)oxan‐2‐yl] 4‐hydroxybenzoate (PubChem CID: 14132341), Creoside III (PubChem CID: 101843543), 2‐(beta‐d‐glucopyranosyloxy)‐1‐(4‐hydroxyphenyl)ethanone (PubChem CID: 101929527), 6‐O‐(e)‐p‐coumaroyl glucopyranose (PubChem CID: 10087731), Picein (PubChem CID: 92123), and 2‐(hydroxymethyl)‐6‐[(4‐hydroxyphenyl)methoxy]oxane‐3,4,5‐triol (PubChem CID: 75311275).

Figure 7Interactions between Salidroside and its analogs with GSDMD. (A) Molecular clustering of 17 key components of HJT. (B) LibDockScore of salidroside and its analogs docking with GSDMD. (C) MOE analysis of the docking binding energies between salidroside and its analogs with GSDMD. (D) Docking binding energies between salidroside and its analogs and GSDMD based on AutoDock analysis. (E) 3D structure of the docking between salidroside and GSDMD. (F) 2D structure of the docking between salidroside and GSDMD.

**Figure:**
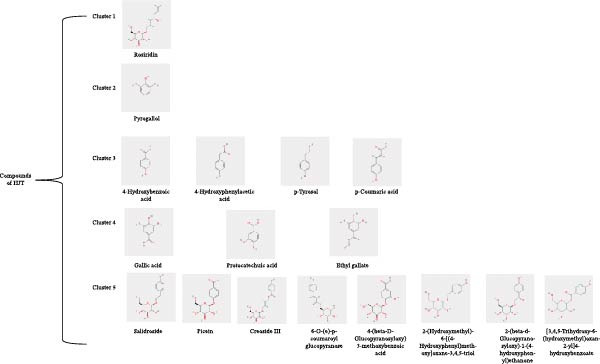
(A)

**Figure:**
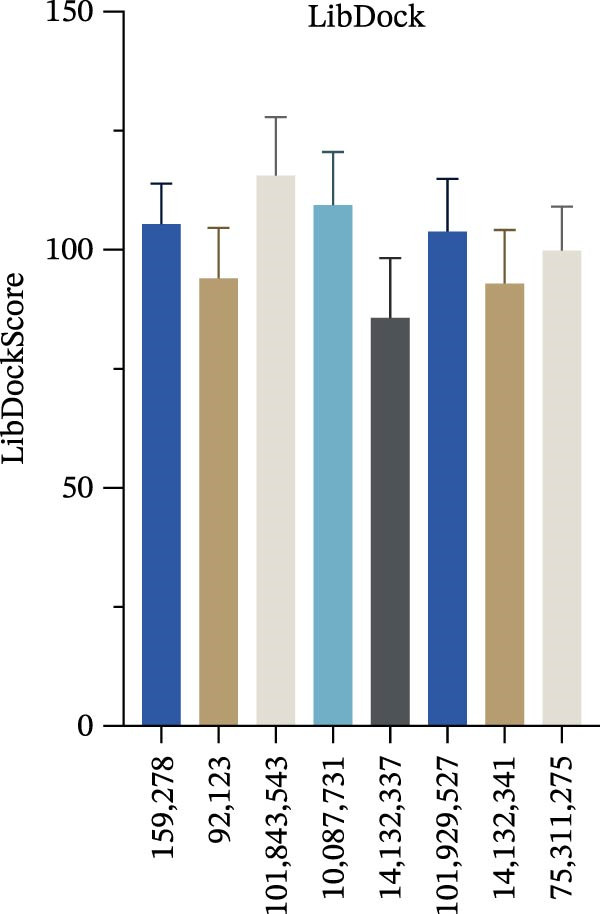
(B)

**Figure:**
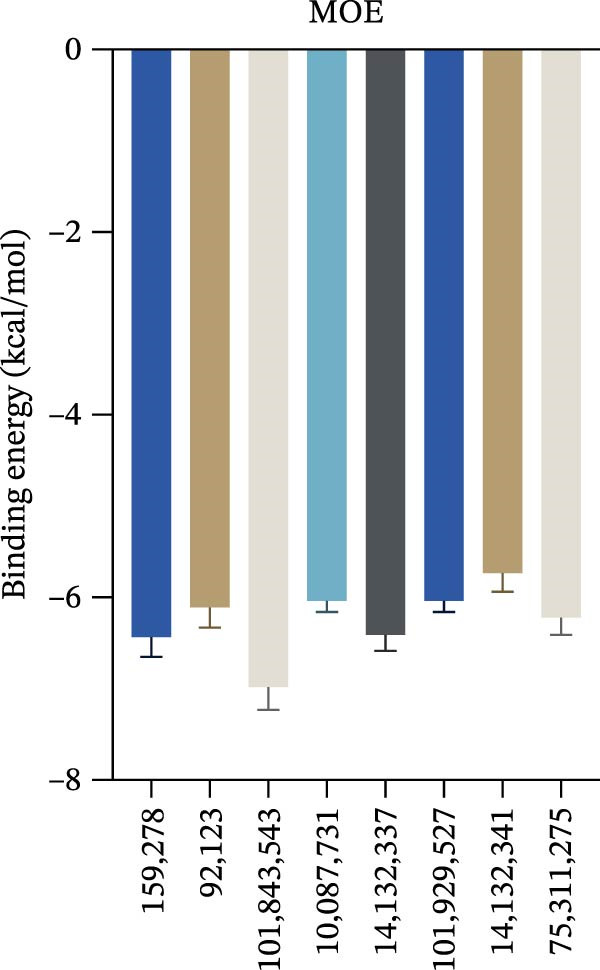
(C)

**Figure:**
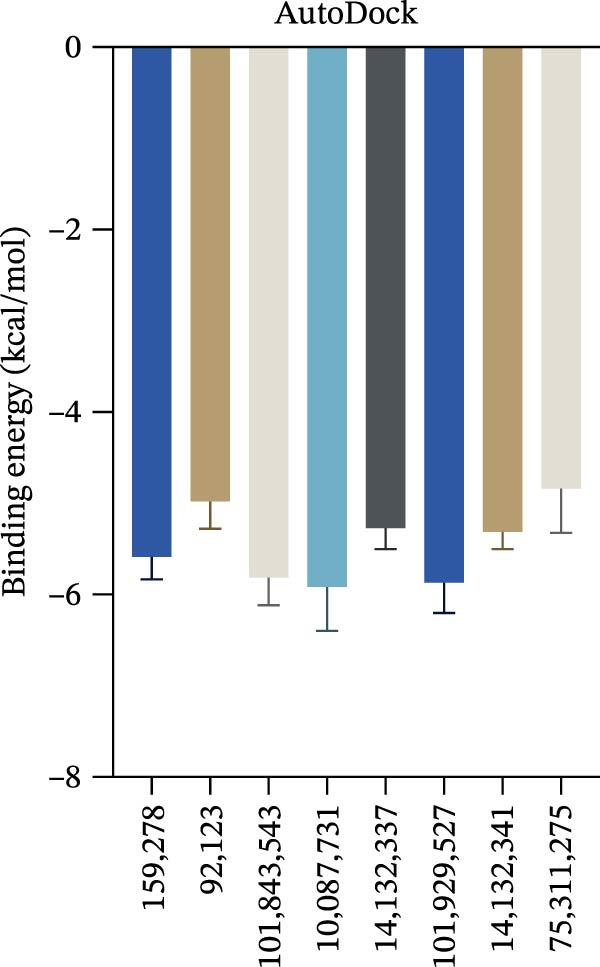
(D)

**Figure:**
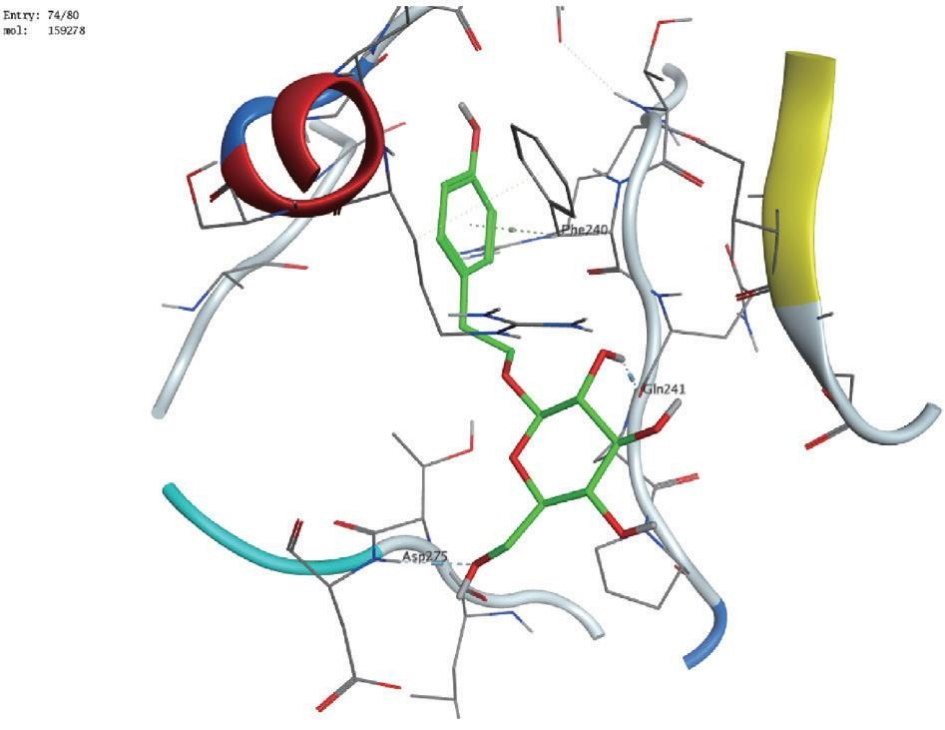
(E)

**Figure:**
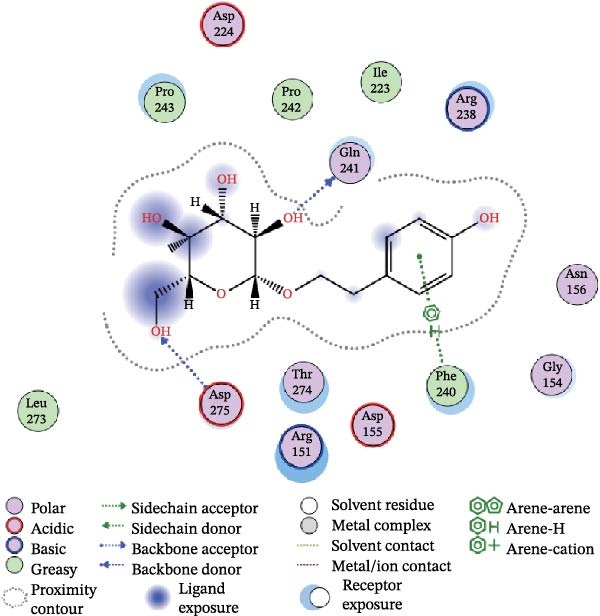
(F)

A small molecule library was constructed based on salidroside (PubChem CID: 159278) and the aforementioned seven small molecules, and molecular docking was performed with the GSDMD protein using three molecular docking methods. LibDock molecular docking results showed that Creoside III and 6‐O‐(e)‐p‐coumaroyl glucopyranose scored higher (Figure 7B, Table S4). MOE molecular docking results showed that salidroside and Creoside III have lower binding energies (Figure 7C, Table S5). AutoDock molecular docking results indicated that Creoside III and 6‐O‐(e)‐p‐coumaroyl glucopyranose have lower binding energies (Figure 7D, Table S6).This suggests that HJT contains salidroside‐related analogs that can bind better to GSDMD. Additionally, MOE molecular docking results indicate that salidroside can form hydrogen bonds with the 275th amino acid of GSDMD through its glycoside moiety, with a docking binding energy of −6.50 kacl/mol (Figure 7e,f). Furthermore, the other seven analogs can also form hydrogen bonds with GSDMD near the docking site (Figure S8A–G). This suggests that HJT may have a stronger inhibitory effect on GSDMD than salidroside.

### 3.8. Experimental Validation of HJT and Salidroside

To further validate the therapeutic effects of HJT and salidroside, treatment experiments were conducted using an animal asthma model. On days 15, 17, 19, 21, and 23 of the experiment, mice in each group were administered the corresponding drugs 30 min before each ozone exposure. All single doses were fully dissolved in a drug solution at a concentration of 10 μL/g based on the body weight of the experimental mice and administered via intraperitoneal injection for a total of 5 days (Figure 8A). To establish a positive control, this study also used common pyroptosis inhibitors, MCC950 and VX765, to verify whether inhibiting the pyroptosis pathway could mitigate the effects of ozone exposure. MCC950 is an effective specific inhibitor of NLRP3, while VX765 is a selective inhibitor of caspase‐1. HE staining (Figure 8B) results showed that compared with the G3 group, the G4–G7 groups exhibited alleviated airway epithelial damage, intra‐airway mucus secretion, and peri‐airway inflammatory cell infiltration, with reduced airway damage and inflammation. Additionally, compared with the G3 group, the staining intensity and extent of α‐SMA immunohistochemistry (Figure 8C) in the airway surrounding the G4–G7 groups were significantly reduced, and airway structural changes were improved. In the western blot experiment (Figure 8D), compared with the G3 group, the expression of NLRP3, ccaspase‐1, and cGSDMD was significantly downregulated in the G4–G7 groups, and statistical significance was confirmed using Image J and grayscale analysis (Figure S9). Immunofluorescence analysis showed that in OVA‐induced asthma mouse lung tissue, compared with the G3 group, the expression of GSDMD (Figure 8E, Figure S10) and NLRP3 (Figure 8F, Figure S11) was also significantly downregulated in the G6 and G7 groups. TUNEL staining was used to detect pyroptosis in tissues. In this study, TUNEL staining (Figure 8G, Figure S12) revealed that compared with the G3 group, HJT and salidroside significantly downregulated TUNEL expression in the G6 and G7 groups, respectively. Notably, immunofluorescence experiments demonstrated that HJT exhibited stronger therapeutic effects compared to salidroside. This suggests that HJT may serve as a therapeutic agent for patients with asthma exacerbated by ozone exposure.

Figure 8Pharmacological validation of HJT and salidroside. (A) Drug treatment process in the asthma model. (B) Expression of cGSDMD, NLRP3, and ccaspase in different drug treatment groups. (C) HE staining results in different drug treatment groups. (D) Expression of α‐SMA in the same drug treatment group. (E) Analysis of GSDMD immunofluorescence intensity in different drug treatment groups using Image J. (F) Analysis of NLRP3 immunofluorescence intensity in different drug treatment groups using Image J. (G) Analysis of TUNEL intensity in different drug treatment groups using Image J.  ^∗^ *p* < 0.05;  ^∗∗^ *p* < 0.01;  ^∗∗∗^ *p* < 0.001;  ^∗∗∗∗^ *p* < 0.0001.

**Figure:**
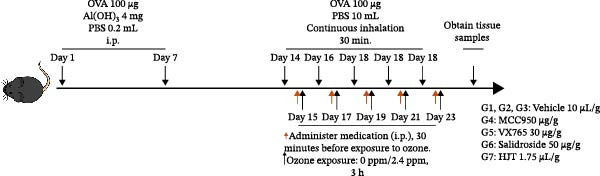
(A)

**Figure:**
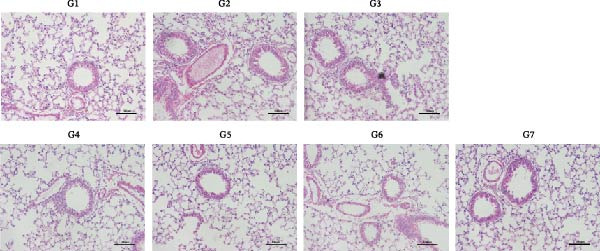
(B)

**Figure:**
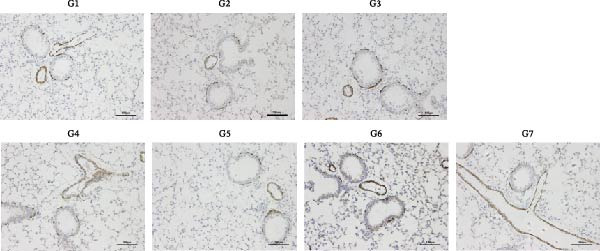
(C)

**Figure:**
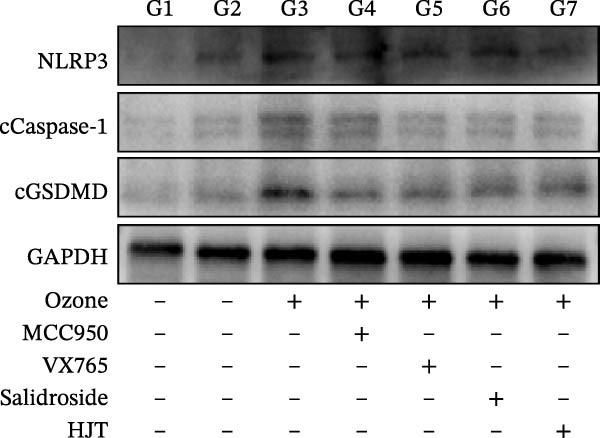
(D)

**Figure:**
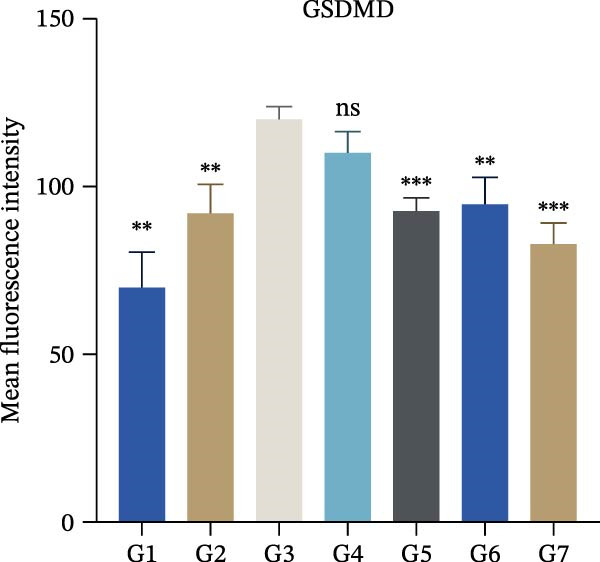
(E)

**Figure:**
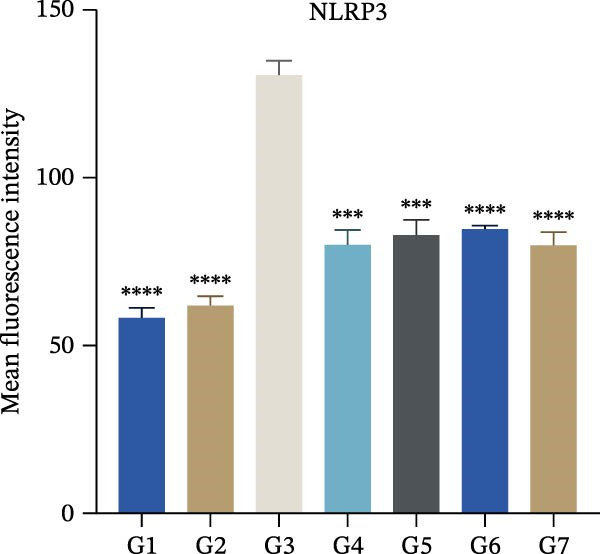
(F)

**Figure:**
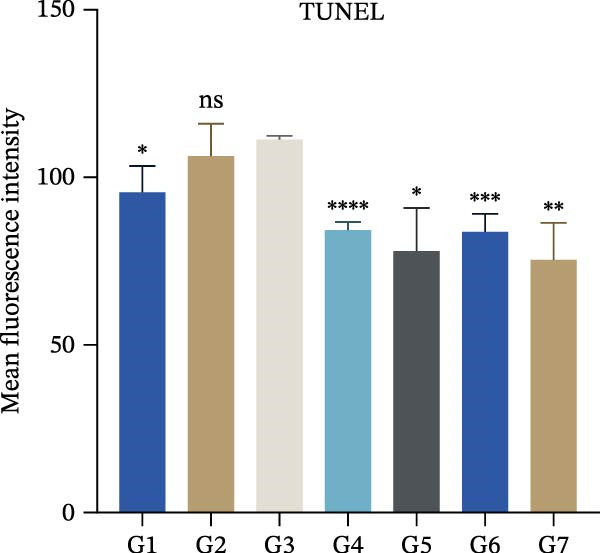
(G)

## 4. Discussion

Pyroptosis is a type of programmed cell death activated by inflammatory caspases, characterized by cell membrane perforation, release of cellular contents, and intense inflammatory responses. Unlike apoptosis, pyroptosis depends on the mediation of the gasdermin protein family (GSDMD and GSDME) and is accompanied by the secretion of pro‐inflammatory factors (IL1β and IL18). In recent years, therapeutic strategies targeting pyroptosis have shown potential in various fields, including cancer, infectious diseases, autoimmune diseases, and neurodegenerative diseases [28–30]. The core mechanism of pyroptosis involves the activation of the inflammasome and the cleavage of gasdermin proteins. Pyroptosis serves as both a response to external stimuli and a new therapeutic target, such as chemotherapy drugs that activate caspase‐3 to cleave GSDME, converting apoptosis into pyroptosis and thereby inhibiting tumor growth [31–33].

Studies have shown that in rheumatoid arthritis and inflammatory bowel disease, abnormal activation of the NLRP3 inflammasome leads to excessive secretion of IL1β. Clinical studies indicate that anti‐IL1β antibodies can alleviate symptoms but cannot block pyroptosis itself. Directly targeting GSDMD with small‐molecule inhibitors that covalently modify Cys191 of GSDMD to inhibit pore formation significantly alleviates colitis in IBD models [31]. Additionally, inhibitors targeting caspase‐1 demonstrate antipyroptotic and anti‐inflammatory effects in psoriasis models. In Alzheimer’s disease, β‐amyloid (Aβ) activates NLRP3 inflammasomes in microglia, leading to neuronal pyroptosis. Inhibiting NLRP3 or GSDMD reduces Aβ deposition and cognitive decline. For example, MCC950 significantly reduced IL1β levels and improved memory deficits in an AD mouse model [34]. In Parkinson’s disease (PD), α‐synuclein aggregation activates the pyroptosis pathway, and inhibitors targeting caspase‐1 demonstrate neuroprotective potential. In myocardial ischemia‐reperfusion injury, mitochondrial DAMPs activate the NLRP3 inflammasome, inducing pyroptosis in cardiomyocytes. Animal studies have shown that GSDMD gene knockout or inhibitors reduce infarct size [35]. Additionally, pyroptosis in macrophages within atherosclerotic plaques promotes plaque instability, and targeting GSDMD may emerge as a new therapeutic direction.

Salidroside and HJT are commonly used in the treatment and research of heart injury‐related diseases and have been shown to have antipyroptosis effects by inhibiting the activation of the pyroptosis pathway through multiple targets. Recent studies have found that salidroside can inhibit airway remodeling in mice through the NF‐κB signaling pathway [36] and regulate the expression of related cytokines, such as IL‐17, IL‐23, IL‐10, and TGF‐β1, thereby inhibiting inflammation. The mechanism by which salidroside inhibits airway inflammation in acute asthma mice is associated with reduced expression of the NLRP3 inflammasome [37, 38]. This study demonstrated that salidroside and HJT can multitarget inhibit the pyroptosis pathway to downregulate GSDMD expression, alleviating airway inflammation and structural changes in ozone‐induced OVA asthma mice, and may serve as a potential therapeutic strategy for treating ozone‐induced asthma in clinical practice.

## Author Contributions

All authors are solely responsible for the content and writing of the manuscript. The study’s design, data collection and analysis, article preparation, and manuscript revision all benefited greatly from the efforts of all authors.

## Funding

This work was supported by the National Natural Science Foundation of China (Grant 82170018).

## Disclosure

There was no person or third‐party service involved in the research or manuscript preparation.

## Consent

This study has been approved by the Research Ethics Committee of the Second Hospital of Hebei Medical University (Number 2024‐AE402).

## Conflicts of Interest

The authors declare no conflicts of interest.

## Supporting Information

Additional supporting information can be found online in the Supporting Information section.

## Supporting information


**Supporting Information** The STROBE‐MR checklist was shown in File S1. The ARRIVE guidelines 2.0 was shown in File S2. The results of predictive models based on other asthma‐related tissue data were shown in File S3, including blood, nasal epithelium, and PBMCs. Figures S1–S12 were shown in supporting information figure with figure legend. Tables S1–S6 were shown in Supporting Information Table.

## Data Availability

The data that support the findings of this study are available from the corresponding author (Xixin Yan) upon reasonable request.
