# Supplementary material for: Salidroside and Hongjingtian Injection Inhibit the Onset and Progression of Asthma via Pyroptosis in the Ozone‐Exposed Inflammation Environment
Source: Mediators Inflamm. 2026 May 7;2026:9618148. doi: 10.1155/mi/9618148 (PMC13150436; doi:10.1155/mi/9618148)
Supplement: Supplementary file 1 — Supporting Information The STROBE‐MR checklist was shown in File S1. The ARRIVE guidelines 2.0 was shown in File S2. The results of predictive models based on other asthma‐related tissue data were shown in File S3, including blood, nasal epithelium, and PBMCs. Figures S1–S12 were shown in supporting information figure with figure legend. Tables S1–S6 were shown in Supporting Information Table. [file MI-2026-9618148-s001.zip › Supplementary Figure.pptx]

## Slide 1
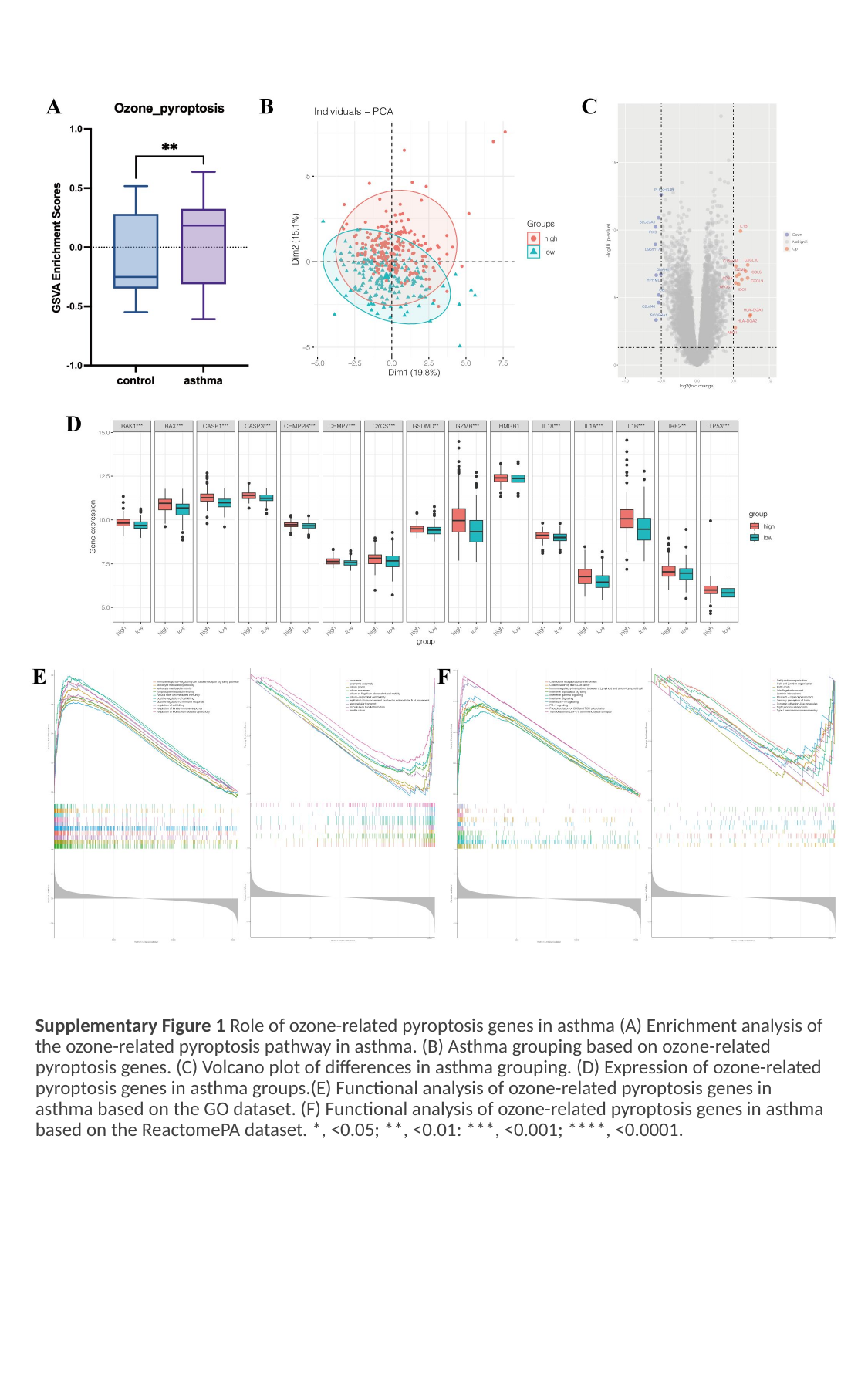

Supplementary Figure 1 Role of ozone-related pyroptosis genes in asthma (A) Enrichment analysis of the ozone-related pyroptosis pathway in asthma. (B) Asthma grouping based on ozone-related pyroptosis genes. (C) Volcano plot of differences in asthma grouping. (D) Expression of ozone-related pyroptosis genes in asthma groups.(E) Functional analysis of ozone-related pyroptosis genes in asthma based on the GO dataset. (F) Functional analysis of ozone-related pyroptosis genes in asthma based on the ReactomePA dataset. *, <0.05; **, <0.01: ***, <0.001; ****, <0.0001.

## Slide 2
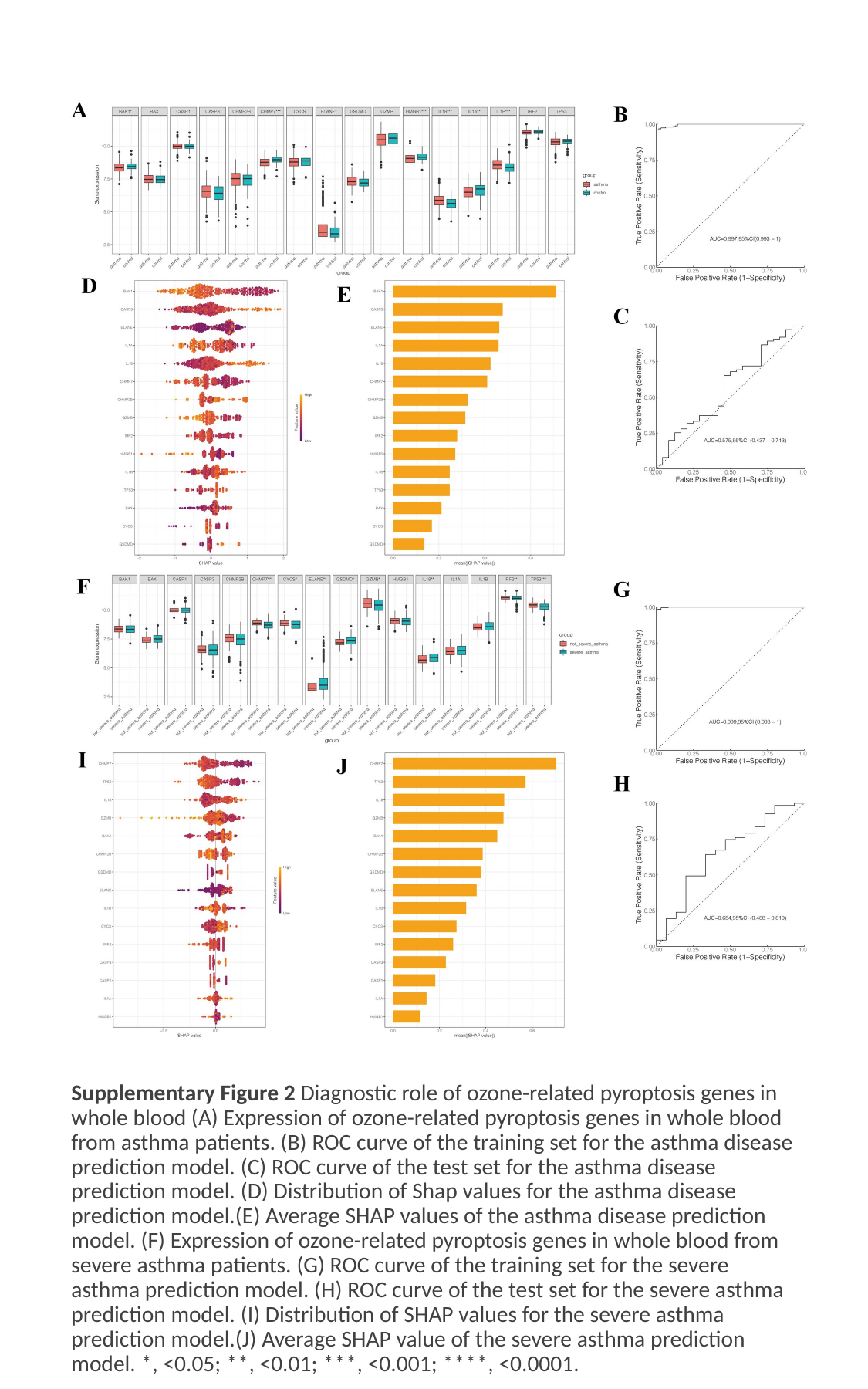

Supplementary Figure 2 Diagnostic role of ozone-related pyroptosis genes in whole blood (A) Expression of ozone-related pyroptosis genes in whole blood from asthma patients. (B) ROC curve of the training set for the asthma disease prediction model. (C) ROC curve of the test set for the asthma disease prediction model. (D) Distribution of Shap values for the asthma disease prediction model.(E) Average SHAP values of the asthma disease prediction model. (F) Expression of ozone-related pyroptosis genes in whole blood from severe asthma patients. (G) ROC curve of the training set for the severe asthma prediction model. (H) ROC curve of the test set for the severe asthma prediction model. (I) Distribution of SHAP values for the severe asthma prediction model.(J) Average SHAP value of the severe asthma prediction model. *, <0.05; **, <0.01; ***, <0.001; ****, <0.0001.

## Slide 3
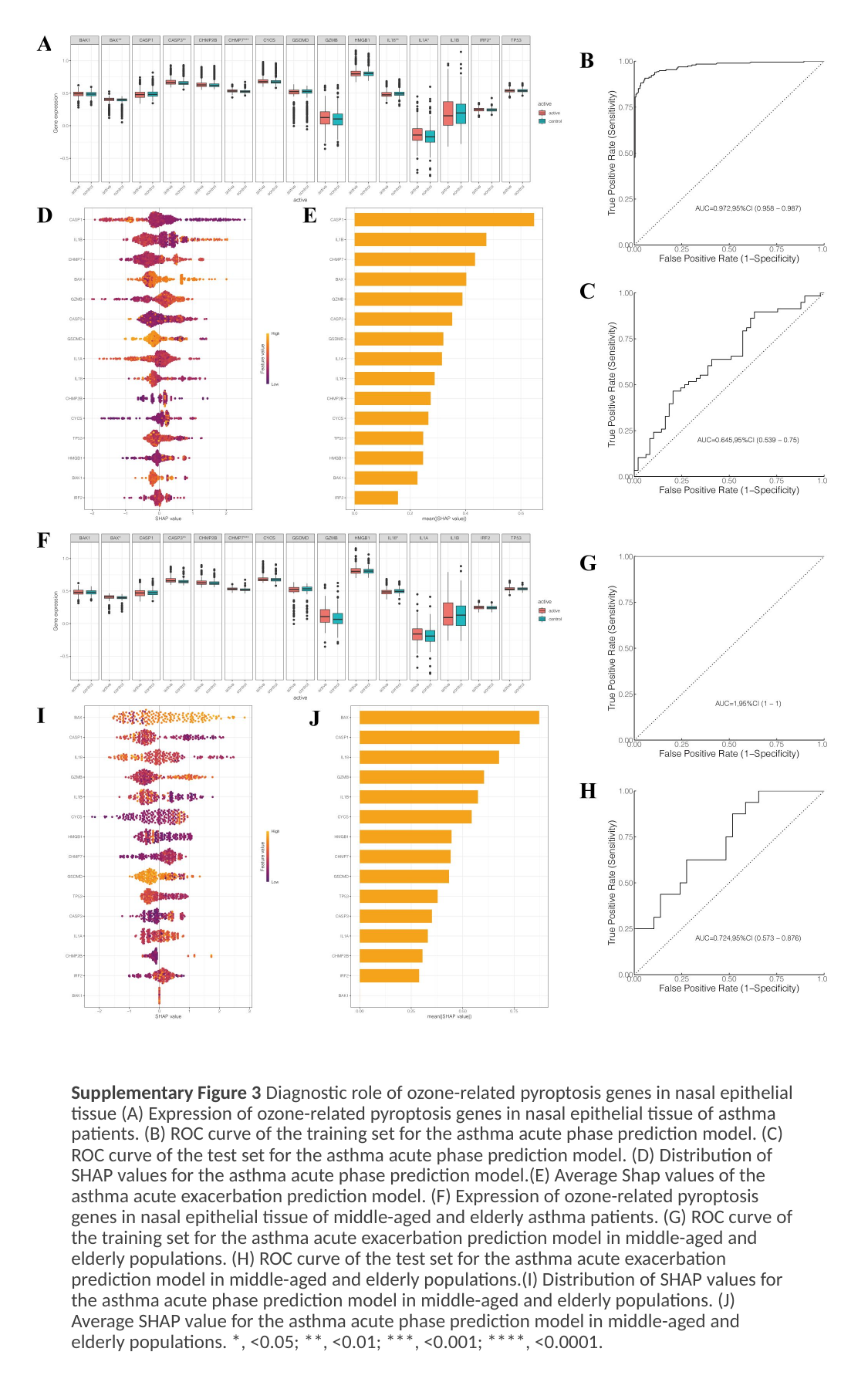

Supplementary Figure 3 Diagnostic role of ozone-related pyroptosis genes in nasal epithelial tissue (A) Expression of ozone-related pyroptosis genes in nasal epithelial tissue of asthma patients. (B) ROC curve of the training set for the asthma acute phase prediction model. (C) ROC curve of the test set for the asthma acute phase prediction model. (D) Distribution of SHAP values for the asthma acute phase prediction model.(E) Average Shap values of the asthma acute exacerbation prediction model. (F) Expression of ozone-related pyroptosis genes in nasal epithelial tissue of middle-aged and elderly asthma patients. (G) ROC curve of the training set for the asthma acute exacerbation prediction model in middle-aged and elderly populations. (H) ROC curve of the test set for the asthma acute exacerbation prediction model in middle-aged and elderly populations.(I) Distribution of SHAP values for the asthma acute phase prediction model in middle-aged and elderly populations. (J) Average SHAP value for the asthma acute phase prediction model in middle-aged and elderly populations. *, <0.05; **, <0.01; ***, <0.001; ****, <0.0001.

## Slide 4
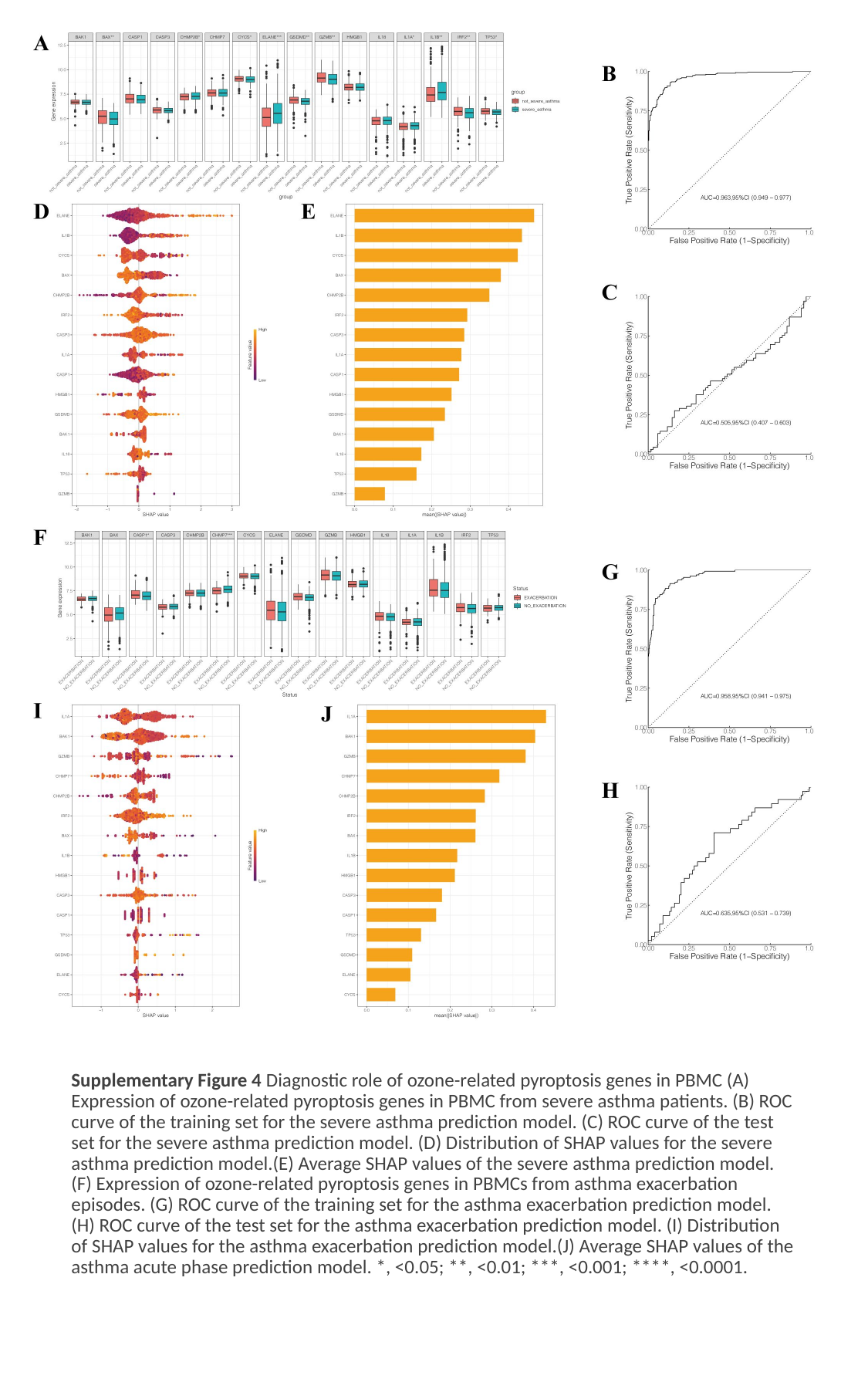

Supplementary Figure 4 Diagnostic role of ozone-related pyroptosis genes in PBMC (A) Expression of ozone-related pyroptosis genes in PBMC from severe asthma patients. (B) ROC curve of the training set for the severe asthma prediction model. (C) ROC curve of the test set for the severe asthma prediction model. (D) Distribution of SHAP values for the severe asthma prediction model.(E) Average SHAP values of the severe asthma prediction model. (F) Expression of ozone-related pyroptosis genes in PBMCs from asthma exacerbation episodes. (G) ROC curve of the training set for the asthma exacerbation prediction model. (H) ROC curve of the test set for the asthma exacerbation prediction model. (I) Distribution of SHAP values for the asthma exacerbation prediction model.(J) Average SHAP values of the asthma acute phase prediction model. *, <0.05; **, <0.01; ***, <0.001; ****, <0.0001.

## Slide 5
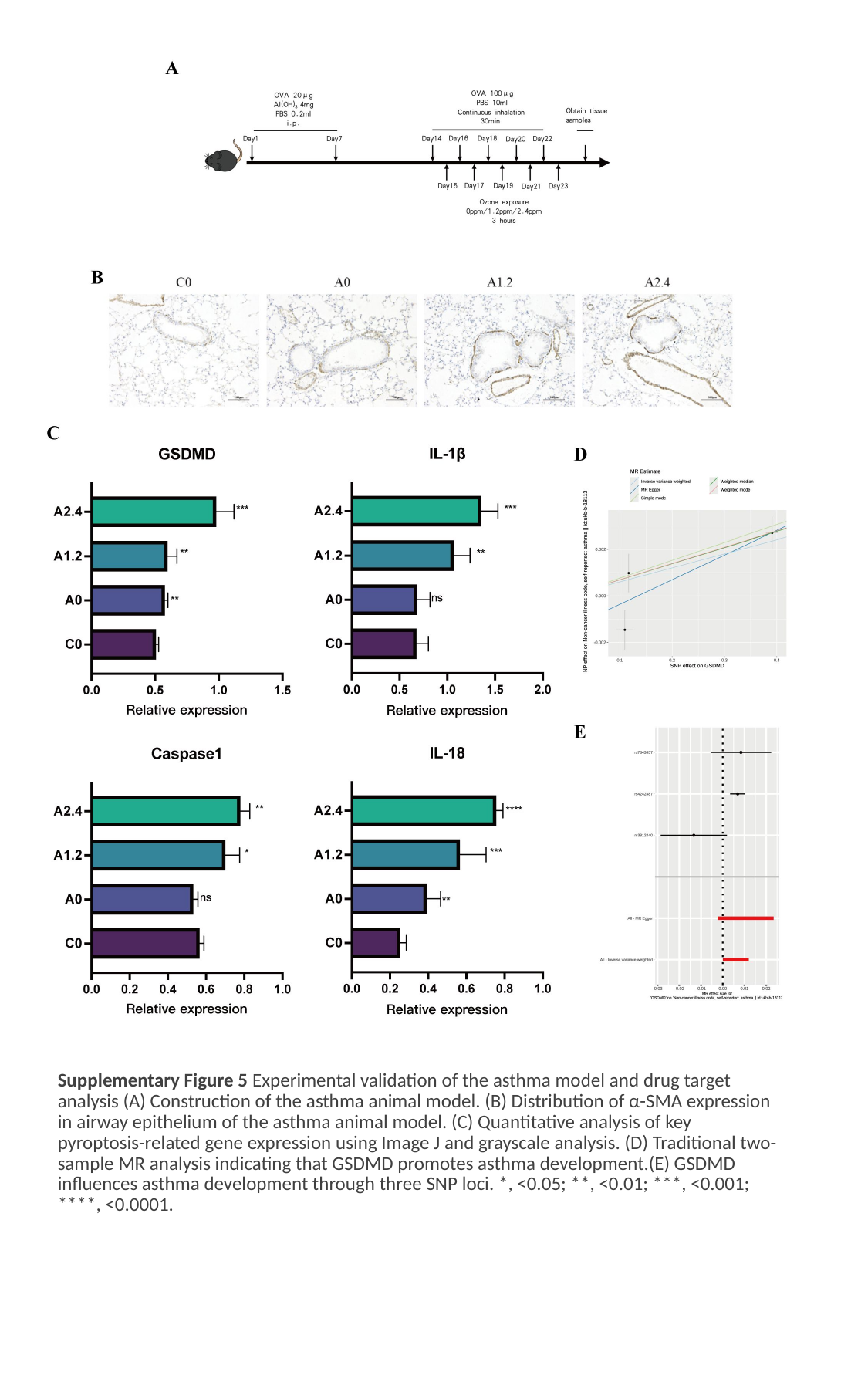

Supplementary Figure 5 Experimental validation of the asthma model and drug target analysis (A) Construction of the asthma animal model. (B) Distribution of α-SMA expression in airway epithelium of the asthma animal model. (C) Quantitative analysis of key pyroptosis-related gene expression using Image J and grayscale analysis. (D) Traditional two-sample MR analysis indicating that GSDMD promotes asthma development.(E) GSDMD influences asthma development through three SNP loci. *, <0.05; **, <0.01; ***, <0.001; ****, <0.0001.

## Slide 6
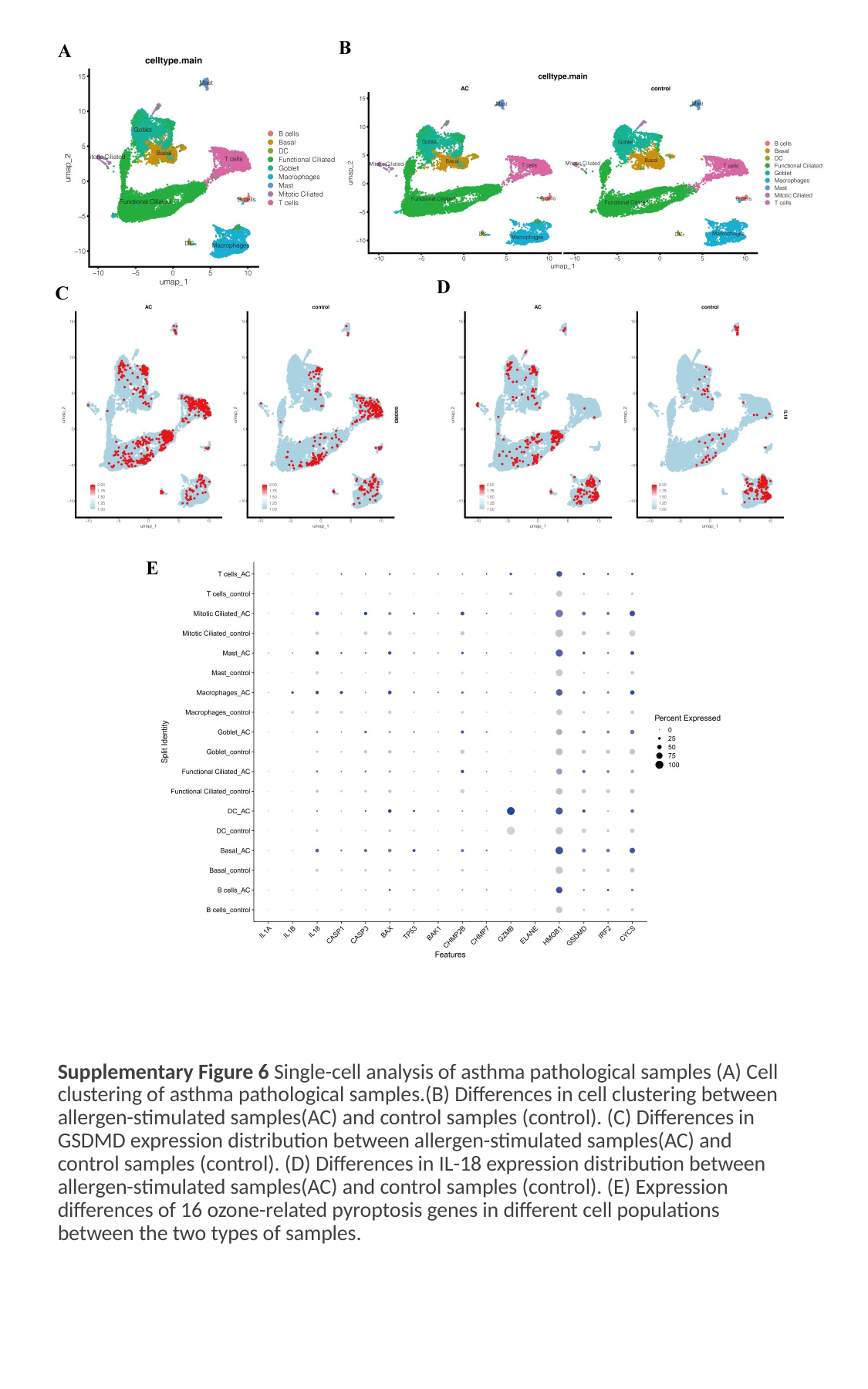

Supplementary Figure 6 Single-cell analysis of asthma pathological samples (A) Cell clustering of asthma pathological samples.(B) Differences in cell clustering between allergen-stimulated samples(AC) and control samples (control). (C) Differences in GSDMD expression distribution between allergen-stimulated samples(AC) and control samples (control). (D) Differences in IL-18 expression distribution between allergen-stimulated samples(AC) and control samples (control). (E) Expression differences of 16 ozone-related pyroptosis genes in different cell populations between the two types of samples.

## Slide 7
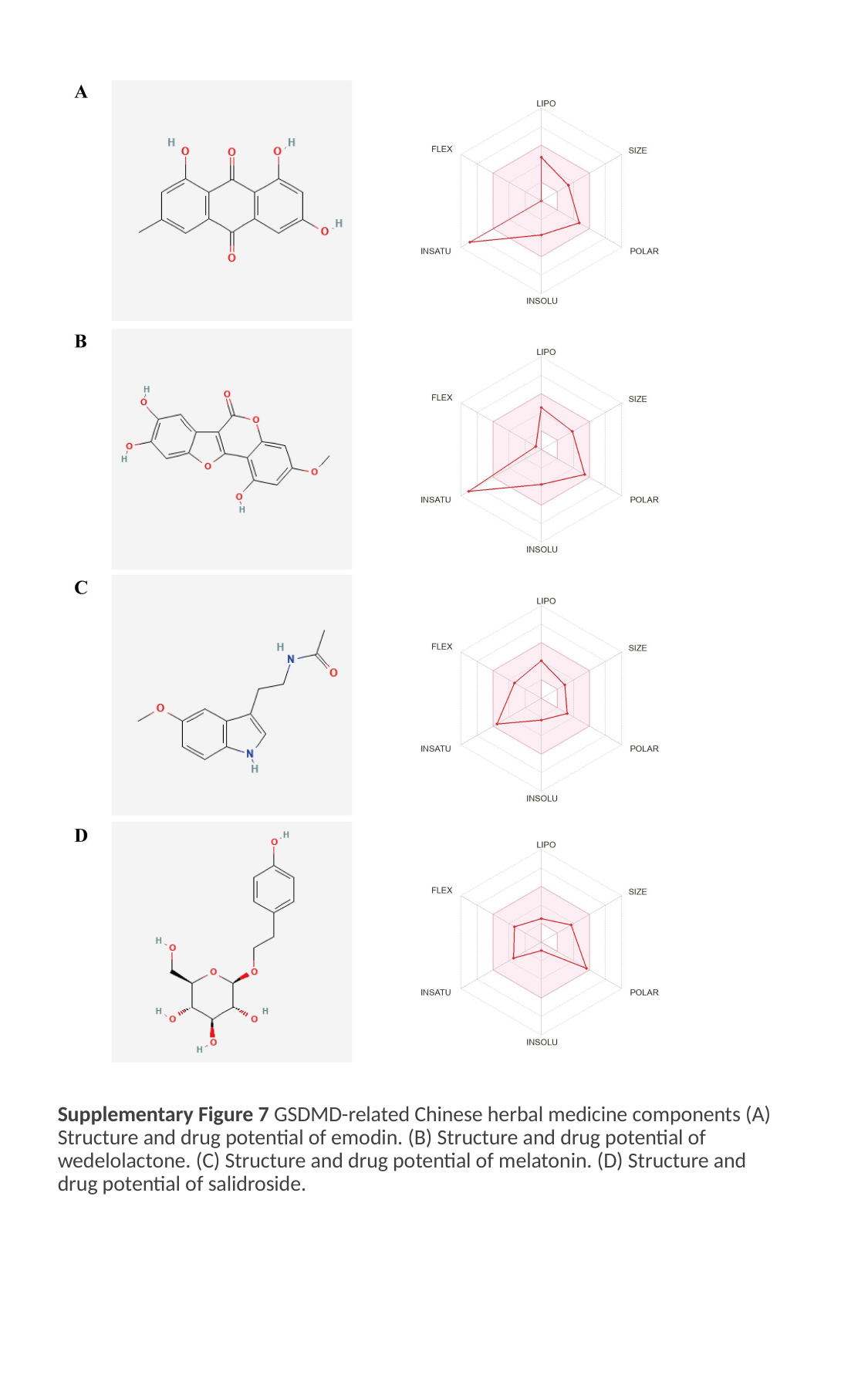

Supplementary Figure 7 GSDMD-related Chinese herbal medicine components (A) Structure and drug potential of emodin. (B) Structure and drug potential of wedelolactone. (C) Structure and drug potential of melatonin. (D) Structure and drug potential of salidroside.

## Slide 8
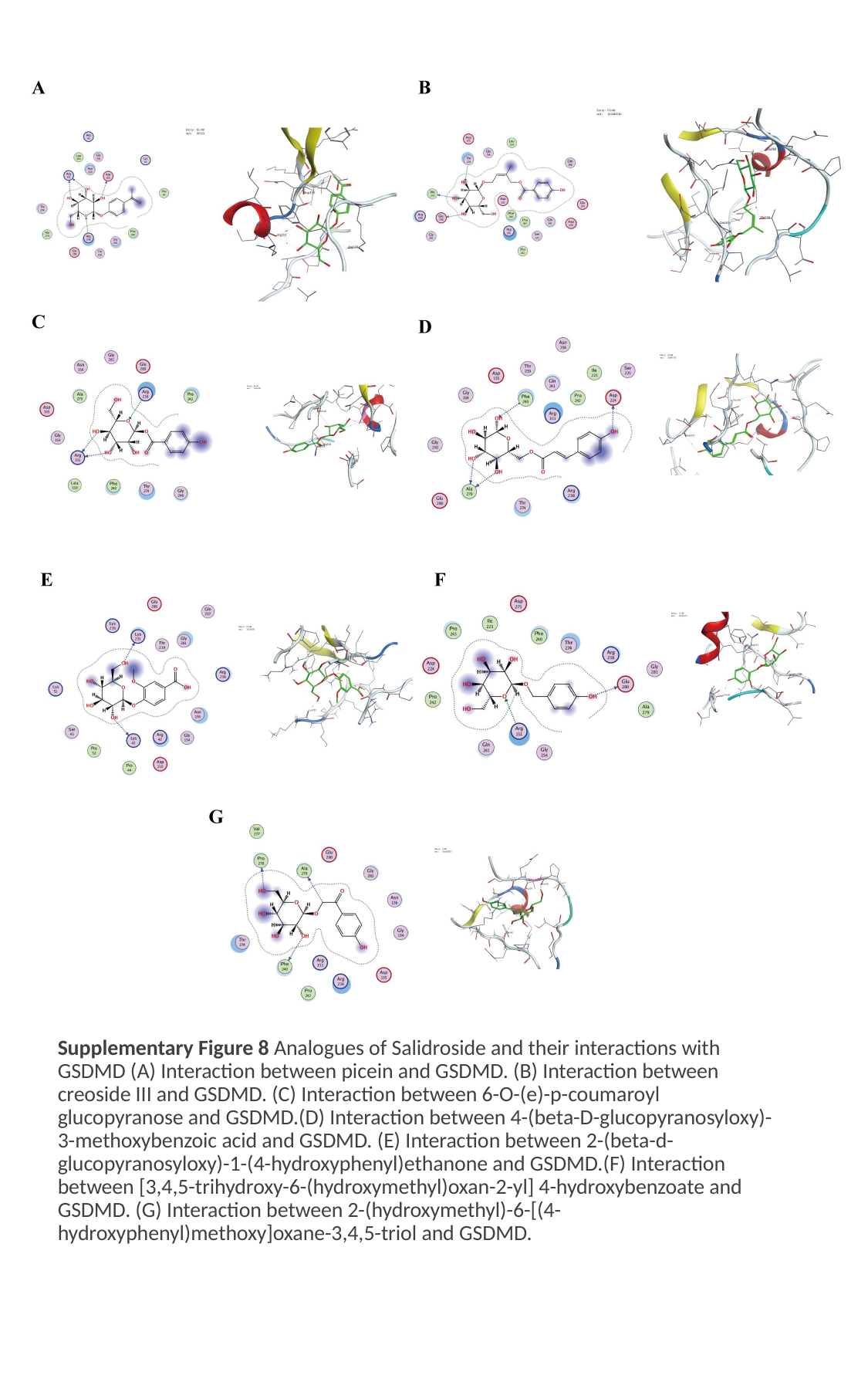

Supplementary Figure 8 Analogues of Salidroside and their interactions with GSDMD (A) Interaction between picein and GSDMD. (B) Interaction between creoside III and GSDMD. (C) Interaction between 6-O-(e)-p-coumaroyl glucopyranose and GSDMD.(D) Interaction between 4-(beta-D-glucopyranosyloxy)-3-methoxybenzoic acid and GSDMD. (E) Interaction between 2-(beta-d-glucopyranosyloxy)-1-(4-hydroxyphenyl)ethanone and GSDMD.(F) Interaction between [3,4,5-trihydroxy-6-(hydroxymethyl)oxan-2-yl] 4-hydroxybenzoate and GSDMD. (G) Interaction between 2-(hydroxymethyl)-6-[(4-hydroxyphenyl)methoxy]oxane-3,4,5-triol and GSDMD.

## Slide 9
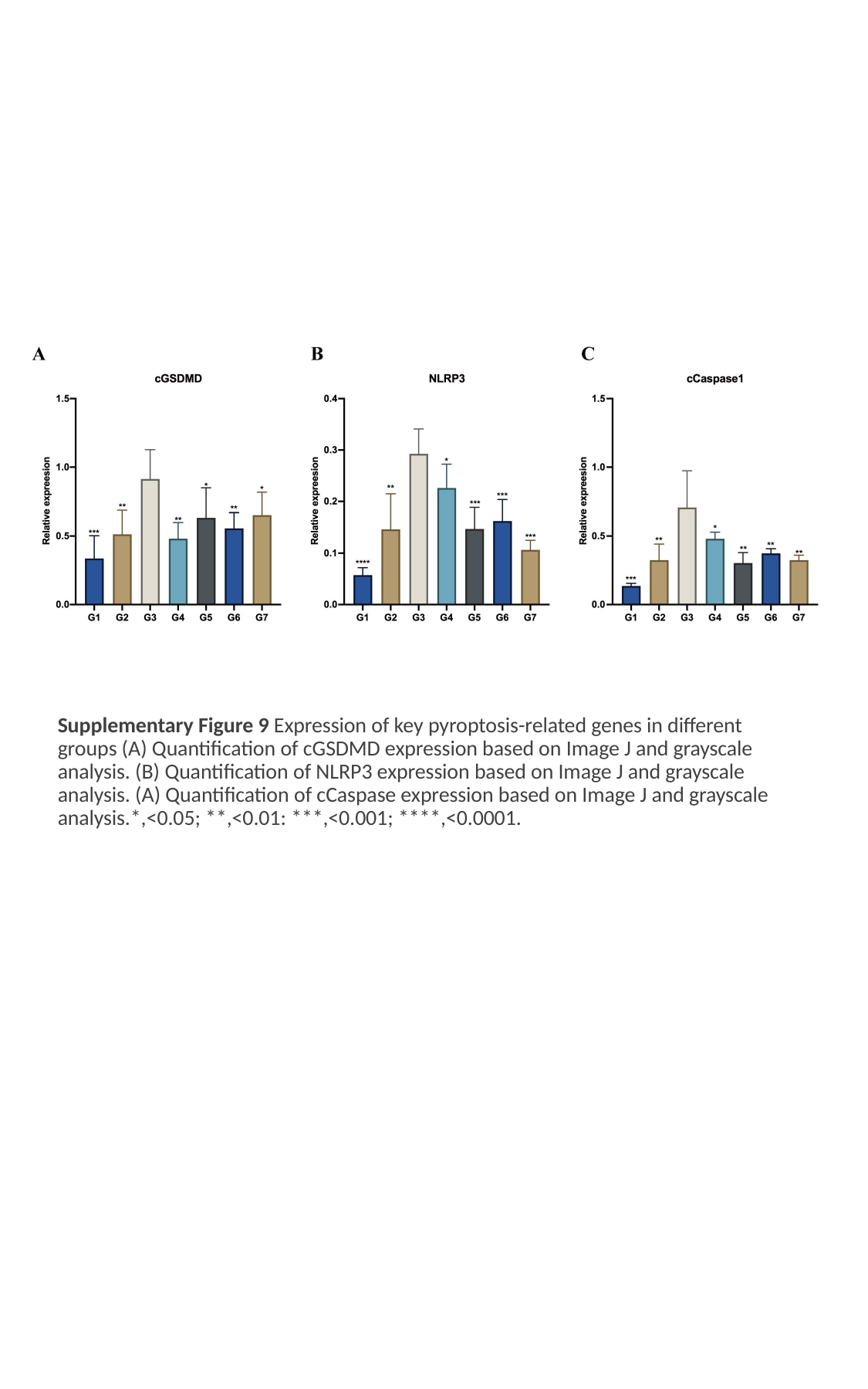

Supplementary Figure 9 Expression of key pyroptosis-related genes in different groups (A) Quantification of cGSDMD expression based on Image J and grayscale analysis. (B) Quantification of NLRP3 expression based on Image J and grayscale analysis. (A) Quantification of cCaspase expression based on Image J and grayscale analysis.*,<0.05; **,<0.01: ***,<0.001; ****,<0.0001.

## Slide 10
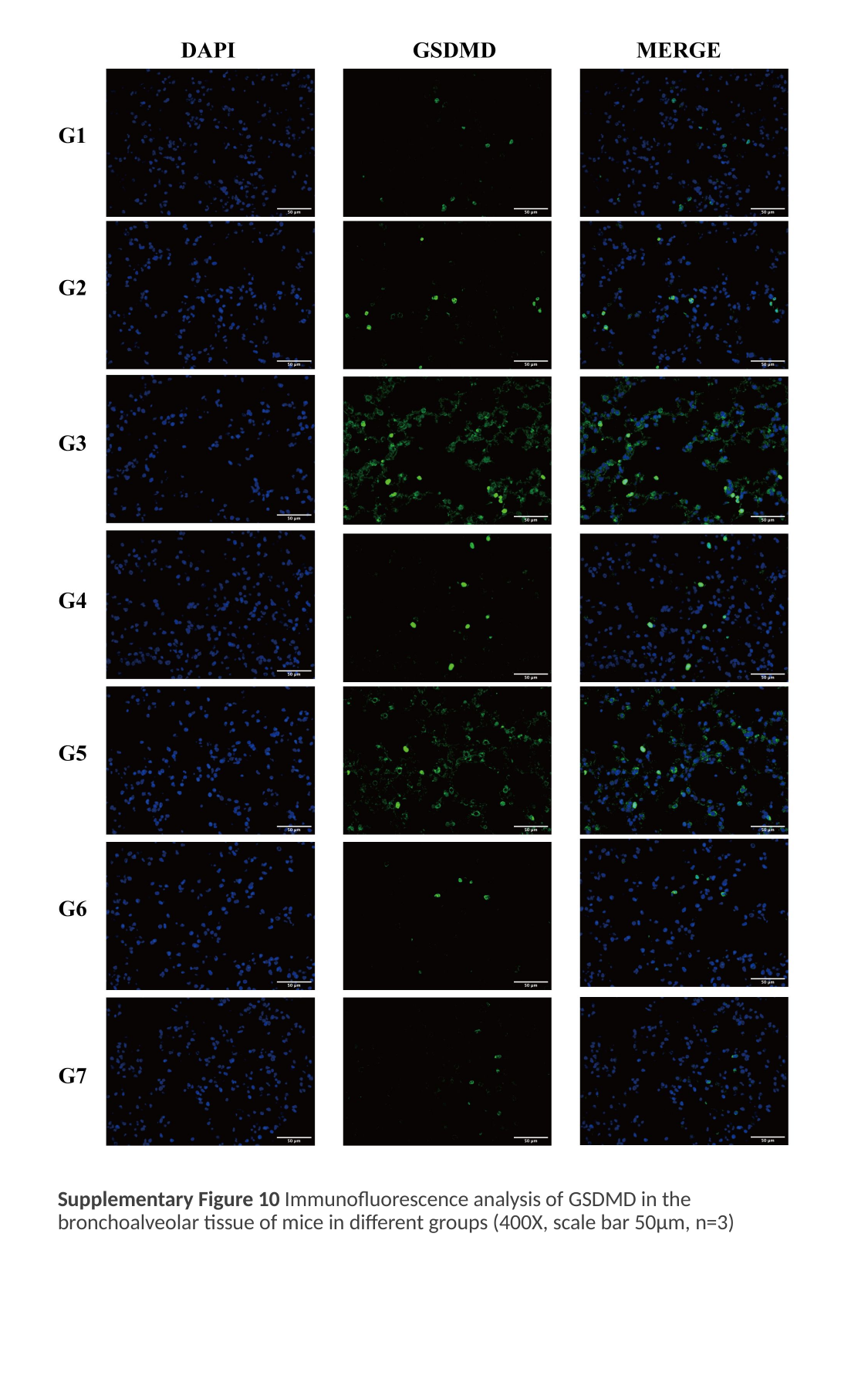

Supplementary Figure 10 Immunofluorescence analysis of GSDMD in the bronchoalveolar tissue of mice in different groups (400X, scale bar 50μm, n=3)

## Slide 11
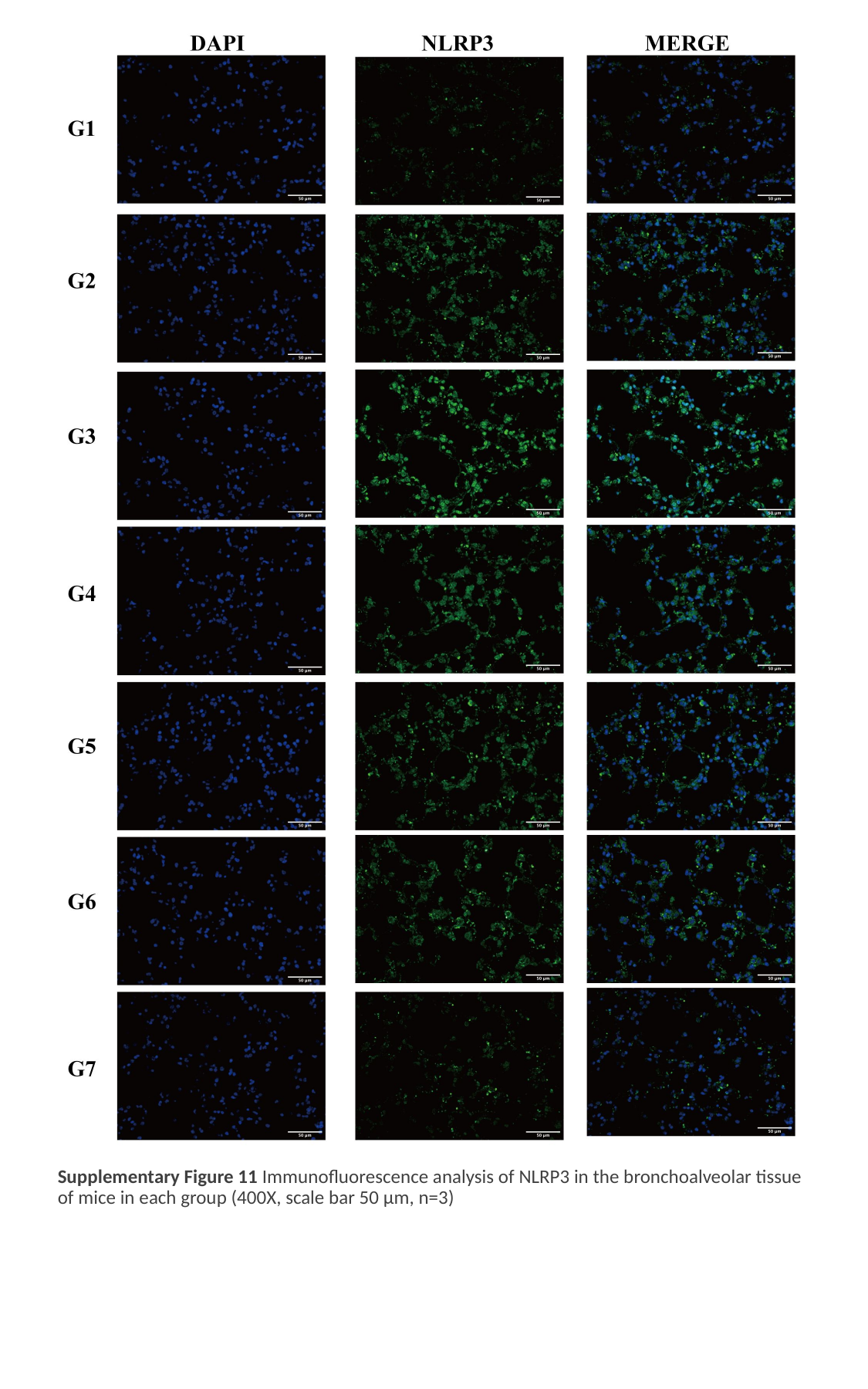

Supplementary Figure 11 Immunofluorescence analysis of NLRP3 in the bronchoalveolar tissue of mice in each group (400X, scale bar 50 μm, n=3)

## Slide 12
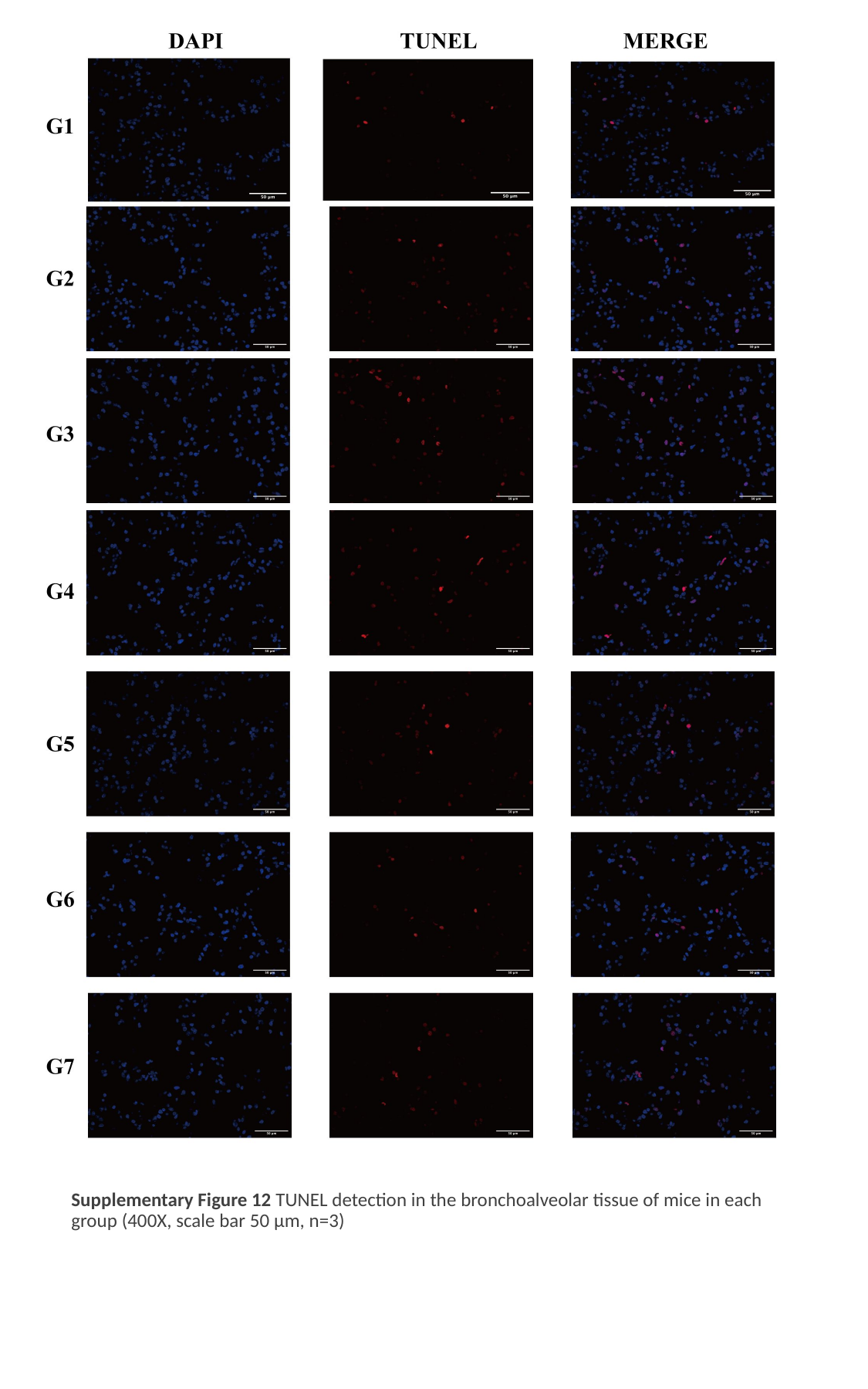

Supplementary Figure 12 TUNEL detection in the bronchoalveolar tissue of mice in each group (400X, scale bar 50 μm, n=3)
