# Supplementary material for: Salidroside and Hongjingtian Injection Inhibit the Onset and Progression of Asthma via Pyroptosis in the Ozone‐Exposed Inflammation Environment
Source: Mediators Inflamm. 2026 May 7;2026:9618148. doi: 10.1155/mi/9618148 (PMC13150436; doi:10.1155/mi/9618148)
Supplement: Supplementary file 1 — Supporting Information The STROBE‐MR checklist was shown in File S1. The ARRIVE guidelines 2.0 was shown in File S2. The results of predictive models based on other asthma‐related tissue data were shown in File S3, including blood, nasal epithelium, and PBMCs. Figures S1–S12 were shown in supporting information figure with figure legend. Tables S1–S6 were shown in Supporting Information Table. [file MI-2026-9618148-s001.zip › Supplementary File 1.docx]

**Supplementary File 1.The STROBE-MR checklist**

**STROBE-MR checklist of recommended items to address in reports of Mendelian randomization studies**^1^ ^2^

| **Item No.** | **Section** | **Checklist item** | **Page No.** | **Relevant text from manuscript** |
| --- | --- | --- | --- | --- |
| 1 | **TITLE and ABSTRACT** | Indicate Mendelian randomization (MR) as the study’s design in the title and/or the abstract if that is a main purpose of the study | 1 | As MR analysis was a secondary method in this study, it does not need to be mentioned in the title. |
|  | **INTRODUCTION** |  |  |  |
| 2 | **Background** | Explain the scientific background and rationale for the reported study. What is the exposure? Is a potential causal relationship between exposure and outcome plausible? Justify why MR is a helpful method to address the study question | 3 | ‌Gasdermin D‌(GSDMD) cleavage-mediated pore formation, and the release of inflammatory cytokines Interleukin-1 beta‌(IL-1β) and Interleukin-18(IL-18), triggering an inflammatory response [13], and is one of the key mechanisms of the body's inflammatory response. |
| 3 | **Objectives** | State specific objectives clearly, including pre-specified causal hypotheses (if any). State that MR is a method that, under specific assumptions, intends to estimate causal effects | 4 | This study aims to analyze the role of pyroptosis in the exacerbation of asthma by ozone exposure using bioinformatics methods and asthma animals models. |
|  | **METHODS** |  |  |  |
| 4 | **Study design and data sources** | Present key elements of the study design early in the article. Consider including a table listing sources of data for all phases of the study. For each data source contributing to the analysis, describe the following: |  |  |
|  | a) | Setting: Describe the study design and the underlying population, if possible. Describe the setting, locations, and relevant dates, including periods of recruitment, exposure, follow-up, and data collection, when available. | 6 | The GWAS data used in this study were obtained from the MRC-IEU, which included 53,598 asthma patients and 409,335 healthy controls. |
|  | b) | Participants: Give the eligibility criteria, and the sources and methods of selection of participants. Report the sample size, and whether any power or sample size calculations were carried out prior to the main analysis | 6 | The GWAS data used in this study were obtained from the MRC-IEU, which included 53,598 asthma patients and 409,335 healthy controls. |
|  | c) | Describe measurement, quality control and selection of genetic variants | 6 | A total of 9,851,867 SNP loci were detected in this study, mainly from European populations. |
|  | d) | For each exposure, outcome, and other relevant variables, describe methods of assessment and diagnostic criteria for diseases | 6 | Based on asthma-related GWAS data and SMR analysis, this study performed Mendelian randomization (MR) analysis on asthma.Two methods were employed in this study: SMR analysis and eQTL analysis. The eQTL analysis was conducted using GWAS data and eQTL data from 16 ozone-related pyroptosis genes.The eQTL data from 16 ozone-related pyroptosis genes were obtained from the eQTLGen Database. |
|  | e) | Provide details of ethics committee approval and participant informed consent, if relevant |  | The data was from Databases. |
| 5 | **Assumptions** | Explicitly state the three core IV assumptions for the main analysis (relevance, independence and exclusion restriction) as well assumptions for any additional or sensitivity analysis |  | The article does not provide a detailed description of the three core assumptions. However, the analytical approach reflects these core assumptions. |
| 6 | **Statistical methods: main analysis** | Describe statistical methods and statistics used |  |  |
|  | a) | Describe how quantitative variables were handled in the analyses (i.e., scale, units, model) | 6 | A total of 9,851,867 SNP loci were detected in this study, mainly from European populations.The eQTL data from 16 ozone-related pyroptosis genes were obtained from the eQTLGen Database. |
|  | b) | Describe how genetic variants were handled in the analyses and, if applicable, how their weights were selected | 6 | The reference coefficient used for SMR analysis is the P-value (PSMR=5×10-6), and the P-value of the HEIDI test (PHEIDI > 0.05, indicating no significant heterogeneity). |
|  | c) | Describe the MR estimator (e.g. two-stage least squares, Wald ratio) and related statistics. Detail the included covariates and, in case of two-sample MR, whether the same covariate set was used for adjustment in the two samples | 6 | SMR analysis, short for Summary-data-based Mendelian Randomization, is a Mendelian randomization method based on summary data [25,26]. |
|  | d) | Explain how missing data were addressed |  | Not applicable |
|  | e) | If applicable, indicate how multiple testing was addressed |  | Not applicable |
| 7 | **Assessment of assumptions** | Describe any methods or prior knowledge used to assess the assumptions or justify their validity | 6 | The reference coefficient used for SMR analysis is the P-value (PSMR=5×10-6), and the P-value of the HEIDI test (PHEIDI > 0.05, indicating no significant heterogeneity). |
| 8 | **Sensitivity analyses and additional analyses** | Describe any sensitivity analyses or additional analyses performed (e.g. comparison of effect estimates from different approaches, independent replication, bias analytic techniques, validation of instruments, simulations) | 6 | The reference coefficient used for SMR analysis is the P-value (PSMR=5×10-6), and the P-value of the HEIDI test (PHEIDI > 0.05, indicating no significant heterogeneity). |
| 9 | **Software and pre-registration** |  |  |  |
|  | a) | Name statistical software and package(s), including version and settings used | 10 | Analysis was performed using R studio (R4.2.1). |
|  | b) | State whether the study protocol and details were pre-registered (as well as when and where) |  | Not applicable |
|  | **RESULTS** |  |  |  |
| 10 | **Descriptive data** |  |  |  |
|  | a) | Report the numbers of individuals at each stage of included studies and reasons for exclusion. Consider use of a flow diagram | 6 | The GWAS data used in this study were obtained from the MRC-IEU, which included 53,598 asthma patients and 409,335 healthy controls. |
|  | b) | Report summary statistics for phenotypic exposure(s), outcome(s), and other relevant variables (e.g. means, SDs, proportions) | 6 | A total of 9,851,867 SNP loci were detected in this study, mainly from European populations.The eQTL data from 16 ozone-related pyroptosis genes were obtained from the eQTLGen Database. |
|  | c) | If the data sources include meta-analyses of previous studies, provide the assessments of heterogeneity across these studies |  | Not applicable |
|  | d) | For two-sample MR:  i.  Provide justification of the similarity of the genetic variant-exposure associations between the exposure and outcome samples  ii.  Provide information on the number of individuals who overlap between the exposure and outcome studies |  | Not applicable |
| 11 | **Main results** |  |  |  |
|  | a) | Report the associations between genetic variant and exposure, and between genetic variant and outcome, preferably on an interpretable scale | 13 | Based on asthma GWAS data and SMR analysis methods, we analyzed asthma-related target genes and identified 754 target genes (Figure 3C).Additionally, based on eQTL analysis, this study found that GSDMD promotes asthma onset (Supplementary Figure 5D) and is associated with three SNP loci (Supplementary Figure 5E). |
|  | b) | Report MR estimates of the relationship between exposure and outcome, and the measures of uncertainty from the MR analysis, on an interpretable scale, such as odds ratio or relative risk per SD difference | 13 | Among these, GSDMD is the target gene significantly associated with asthma among ozone-related pyroptosis genes, with SMR detection showing p = 0.000171 and HEIDI detection p = 0.828. In asthma data, the SNP significantly associated with GSDMD (top-cis eQTL) is rs4874140 (Figure 3D).This locus is located at chromosome 8 at position 144571841, with a mutation rate of 0.1948, and is associated with multiple genes (GSDMD, ZC3H3, and MROH6) (Figure 3E) |
|  | c) | If relevant, consider translating estimates of relative risk into absolute risk for a meaningful time period |  | Not applicable |
|  | d) | Consider plots to visualize results (e.g. forest plot, scatterplot of associations between genetic variants and outcome versus between genetic variants and exposure) |  | Plots from Figure 3 and Supplementary Figure 5. |
| 12 | **Assessment of assumptions** |  |  |  |
|  | a) | Report the assessment of the validity of the assumptions | 13 | Among these, GSDMD is the target gene significantly associated with asthma among ozone-related pyroptosis genes, with SMR detection showing p = 0.000171 and HEIDI detection p = 0.828. |
|  | b) | Report any additional statistics (e.g., assessments of heterogeneity across genetic variants, such as *I^2^*, Q statistic or E-value) | 13 | Among these, GSDMD is the target gene significantly associated with asthma among ozone-related pyroptosis genes, with SMR detection showing p = 0.000171 and HEIDI detection p = 0.828. |
| 13 | **Sensitivity analyses and additional analyses** |  |  |  |
|  | a) | Report any sensitivity analyses to assess the robustness of the main results to violations of the assumptions | 13 | Among these, GSDMD is the target gene significantly associated with asthma among ozone-related pyroptosis genes, with SMR detection showing p = 0.000171 and HEIDI detection p = 0.828. |
|  | b) | Report results from other sensitivity analyses or additional analyses |  | Not applicable |
|  | c) | Report any assessment of direction of causal relationship (e.g., bidirectional MR) |  | Not applicable |
|  | d) | When relevant, report and compare with estimates from non-MR analyses |  | Not applicable |
|  | e) | Consider additional plots to visualize results (e.g., leave-one-out analyses) |  | Not applicable |
|  | **DISCUSSION** |  |  |  |
| 14 | **Key results** | Summarize key results with reference to study objectives | 13 | In summary, based on drug target MR analysis results, GSDMD may be an important target gene in asthma patients and could serve as a potential therapeutic target for asthma. |
| 15 | **Limitations** | Discuss limitations of the study, taking into account the validity of the IV assumptions, other sources of potential bias, and imprecision. Discuss both direction and magnitude of any potential bias and any efforts to address them |  | Not applicable |
| 16 | **Interpretation** |  |  |  |
|  | a) | Meaning: Give a cautious overall interpretation of results in the context of their limitations and in comparison with other studies | 18 | In recent years, therapeutic strategies targeting pyroptosis have shown potential in various fields, including cancer, infectious diseases, autoimmune diseases, and neurodegenerative diseases [28-30]. |
|  | b) | Mechanism: Discuss underlying biological mechanisms that could drive a potential causal relationship between the investigated exposure and the outcome, and whether the gene-environment equivalence assumption is reasonable. Use causal language carefully, clarifying that IV estimates may provide causal effects only under certain assumptions | 18 | Pyroptosis is a type of programmed cell death activated by inflammatory caspases, characterized by cell membrane perforation, release of cellular contents, and intense inflammatory responses. Unlike apoptosis, pyroptosis depends on the mediation of the gasdermin protein family (GSDMD and GSDME) and is accompanied by the secretion of pro-inflammatory factors (IL-1β and IL-18). |
|  | c) | Clinical relevance: Discuss whether the results have clinical or public policy relevance, and to what extent they inform effect sizes of possible interventions | 18 | The core mechanism of pyroptosis involves the activation of the inflammasome and the cleavage of gasdermin proteins. Pyroptosis serves as both a response to external stimuli and a new therapeutic target, such as chemotherapy drugs that activate Caspase-3 to cleave GSDME, converting apoptosis into pyroptosis and thereby inhibiting tumor growth [31-33]. |
| 17 | **Generalizability** | Discuss the generalizability of the study results (a) to other populations, (b) across other exposure periods/timings, and (c) across other levels of exposure |  | Not applicable |
|  | **OTHER INFORMATION** |  |  |  |
| 18 | **Funding** | Describe sources of funding and the role of funders in the present study and, if applicable, sources of funding for the databases and original study or studies on which the present study is based | 19 | This work was supported by the National Natural Science Foundation of China (Grant No. 82170018). |
| 19 | **Data and data sharing** | Provide the data used to perform all analyses or report where and how the data can be accessed, and reference these sources in the article. Provide the statistical code needed to reproduce the results in the article, or report whether the code is publicly accessible and if so, where | 20 | The data that support the findings of this study are available from the corresponding author(Xixin Yan) upon reasonable request. |
| 20 | **Conflicts of Interest** | All authors should declare all potential conflicts of interest | 19 | There are no conflicts of interest declared by any of the authors. |

This checklist is copyrighted by the Equator Network under the Creative Commons Attribution 3.0 Unported (CC BY 3.0) license.

1. Skrivankova VW, Richmond RC, Woolf BAR, Yarmolinsky J, Davies NM, Swanson SA, et al. Strengthening the Reporting of Observational Studies in Epidemiology using Mendelian Randomization (STROBE-MR) Statement. JAMA. 2021;under review.

2. Skrivankova VW, Richmond RC, Woolf BAR, Davies NM, Swanson SA, VanderWeele TJ, et al. Strengthening the Reporting of Observational Studies in Epidemiology using Mendelian Randomisation (STROBE-MR): Explanation and Elaboration. BMJ. 2021;375:n2233.
