# Supplementary material for: Salidroside and Hongjingtian Injection Inhibit the Onset and Progression of Asthma via Pyroptosis in the Ozone‐Exposed Inflammation Environment
Source: Mediators Inflamm. 2026 May 7;2026:9618148. doi: 10.1155/mi/9618148 (PMC13150436; doi:10.1155/mi/9618148)
Supplement: Supplementary file 1 — Supporting Information The STROBE‐MR checklist was shown in File S1. The ARRIVE guidelines 2.0 was shown in File S2. The results of predictive models based on other asthma‐related tissue data were shown in File S3, including blood, nasal epithelium, and PBMCs. Figures S1–S12 were shown in supporting information figure with figure legend. Tables S1–S6 were shown in Supporting Information Table. [file MI-2026-9618148-s001.zip › Supplementary File 3.docx]

**Supplementary File 3** The results of predictive models based on other asthma-related tissue data, including blood, nasal epithelium, and PBMCs

**1.Blood**

Gene expression in blood is a common disease biomarker in clinical practice. The blood cohort included 87 healthy controls and 411 asthma patients, among whom 334 were severe asthma patients and 77 were non-severe asthma patients.We found that asthma patients had significantly higher expression of IL-1β and IL-18 in their blood compared to healthy individuals (Supplementary Figure 2A). Using the XGBoost method and gene expression data, we constructed an asthma disease prediction model to predict whether patients have asthma. The ROC curve showed an AUC value of 0.997 (0.993–1) for the training set (Supplementary Figure 2B).A random 20% of the data was used as a test set, yielding an AUC value of 0.575 (0.437–0.713) for the test set (Supplementary Figure 2C). SHAP analysis revealed that IL1A and IL1β have strong diagnostic predictive roles (Supplementary Figure 2E).Among these, CASP3 and IL18 showed a significant increase in SHAP values with increasing gene expression (Supplementary Figure 2D), indicating they are important risk factors for asthma development. We also found that severe asthma patients had higher expression levels of GSDMD and IL18 in their blood compared to non-severe asthma patients (Supplementary Figure 2F).In this study, we also constructed a severe asthma prediction model to predict whether asthma patients are severe asthma. The ROC curve showed that the AUC value of the training set was 0.999 (0.998–1) (Supplementary Figure 2G).A random 20% of the data was used as a test set for validation, showing an AUC value of 0.654 (0.488–0.819) (Supplementary Figure 2H).SHAP analysis revealed that IL18 has a strong diagnostic predictive role (Supplementary Figure 2J). Additionally, as gene expression increases, SHAP values show a significant increase (Supplementary Figure 2I), making it an important risk factor for the development of severe asthma.

**2.Nasal epithelium**

Nasal epithelial tissue is an important component of the respiratory tract. This study utilized nasal epithelial tissue sequencing data from asthma patients to investigate the expression and diagnostic predictive role of 16 ozone-related pyroptosis genes in nasal epithelial tissue. Nasal epithelial tissue was obtained from the GSE240567 cohort, which included 256 acute asthma cases and 297 healthy controls.Additionally, the cohort included 234 middle-aged and elderly individuals, comprising 107 acute asthma patients and 127 healthy controls. In the entire cohort, we found that acute asthma patients exhibited higher expression of BAX and CASP3 in nasal epithelial tissue compared to healthy controls (Supplementary Figure 3A).Using the XGBoost method and gene expression data, we constructed a severe asthma prediction model to predict whether patients have severe asthma. The ROC curve showed an AUC value of 0.972 (0.958–0.987) for the training set (Supplementary Figure 3B).A random 20% of the data was used as a test set for validation, showing an AUC value of 0.645 (0.539–0.75) for the test set (Supplementary Figure 3C). SHAP analysis revealed that CASP1 and IL1β exhibit strong diagnostic predictive roles (Supplementary Figure 3E).Among these, IL1β exhibited a significant increase in SHAP values with increasing gene expression (Supplementary Figure 3D), indicating it is an important risk factor for asthma onset. In the middle-aged and elderly population, we also found that BAX and CASP3 were highly expressed in nasal epithelial tissue of acute asthma patients compared to non-acute phase patients (Supplementary Figure 3F).We constructed an acute asthma prediction model for middle-aged and elderly individuals to predict whether middle-aged asthma patients are in the acute phase. The ROC curve showed an AUC value of 1 (1-1) for the training set (Supplementary Figure 3G). A random 20% of the data was used as the test set for validation, yielding an AUC value of 0.724 (0.573–0.876)(Supplementary Figure 3H). SHAP analysis indicated that CASP1, IL18, and IL1β have strong diagnostic predictive roles (Supplementary Figure 3J). Among these, BAX exhibits increasing SHAP values with rising gene expression (Supplementary Figure 3I), making it an important risk factor for the occurrence of acute asthma in middle-aged and elderly populations.

**3.PBMCs**

PBMCs are an important cell population in blood. This study utilized PBMC sequencing data from asthma patients to detect the expression and diagnostic predictive value of 16 ozone-related pyroptosis genes in whole blood. The cohort included 342 non-severe asthma patients and 343 severe asthma patients. Among these asthma patients, 166 were in the acute phase and 519 were in the non-acute phase.We found that severe asthma patients exhibited higher expression of IL-1β in PBMCs compared to non-severe asthma patients (Supplementary Figure 4A). Using the XGBoost method and gene expression data, we constructed a severe asthma prediction model to predict whether patients have severe asthma. The ROC curve showed an AUC value of 0.963 (0.949–0.977) for the training set(Supplementary Figure 4B). A random 20% of the data was used as a test set, yielding an AUC value of 0.505 (0.407–0.603) for the test set (Supplementary Figure 4C). SHAP analysis indicated that IL-1β has strong diagnostic predictive value (Supplementary Figure 4E).Additionally, SHAP values increase significantly with higher gene expression (Supplementary Figure 4D), indicating that IL1β is an important risk factor for asthma development. We also found that CASP1 is highly expressed in PBMCs during the acute phase compared to the non-acute phase (Supplementary Figure 4F). In this study, we also constructed an acute phase prediction model for asthma to predict whether asthma patients are in the acute phase.The ROC curve showed that the AUC value of the training set was 0.958 (0.941–0.975) (Supplementary Figure 4G). A random 20% of the data was used as the test set for validation, showing an AUC value of 0.635 (0.531–0.739) (Supplementary Figure 4H).SHAP analysis revealed that IL1A and BAK1 exhibit strong diagnostic predictive roles (Supplementary Figure 4J). Additionally, as gene expression increases, SHAP values also increase (Supplementary Figure 4I), indicating that these genes are important risk factors for the onset of asthma acute episodes.
